# Growth Factors as Axon Guidance Molecules: Lessons From *in vitro* Studies

**DOI:** 10.3389/fnins.2021.678454

**Published:** 2021-05-21

**Authors:** Massimo M. Onesto, Caitlin A. Short, Sarah K. Rempel, Timothy S. Catlett, Timothy M. Gomez

**Affiliations:** Neuroscience Training Program and Cell and Molecular Biology Program, Department of Neuroscience, University of Wisconsin–Madison, Madison, WI, United States

**Keywords:** development, dendrite, spine, growth cone, cytoskeleton

## Abstract

Growth cones at the tips of extending axons navigate through developing organisms by probing extracellular cues, which guide them through intermediate steps and onto final synaptic target sites. Widespread focus on a few guidance cue families has historically overshadowed potentially crucial roles of less well-studied growth factors in axon guidance. In fact, recent evidence suggests that a variety of growth factors have the ability to guide axons, affecting the targeting and morphogenesis of growth cones *in vitro.* This review summarizes *in vitro* experiments identifying responses and signaling mechanisms underlying axon morphogenesis caused by underappreciated growth factors.

## Introduction

Throughout organismal development, cell signals activated by extrinsic cues play essential roles in controlling cell fates. For example, by regulating cell survival, differentiation, and morphogenesis, growth factor signaling is crucial for the organogenesis of every tissue in a developing organism. In particular, the central nervous system relies heavily on these signals to assemble intricate neuronal networks. Growth cones at the tips of extending axons probe their surroundings and convert chemosensory signals into mechanical responses that elicit changes in process outgrowth, facilitating attractive and repulsive axon guidance toward target sites. For over three decades researchers have focused on understanding the roles of just a few families of guidance cues in network assembly, yet many more families of growth factors act through similar signaling cascades. Well-studied cues include the Netrin, Semaphorin, Slit, Ephrin, and Wnt family of guidance factors. While these factors have proven to be essential for the guidance and targeting of axons, additional factors likely also play important roles in neural network wiring.

Three lines of evidence support roles for traditional growth factors as axon guidance cues: the expression patterns of ligands and receptors during development, the effects of manipulation of these expression patterns on axon guidance *in vivo*, and analysis of guidance by growth factors in simplified *in vitro* experiments. We have previously reviewed the compelling evidence for roles of growth factors as axon guidance factors *in vivo* (Short et al., 2021). Here we review the endogenous expression patterns and *in vitro* studies that support those *in vivo* experiments. Experiments performed in reduced conditions *in vitro* are powerful as they uncover molecular mechanisms that regulate axon guidance and can test the combinatorial effects of multiple cues which contribute to the diversity of cue effects *in vivo*. Several purified recombinant growth factors affect neuronal survival, differentiation, and neuronal morphogenesis of different classes of developing neurons *in vitro*. Growth factors can act directly to guide neurons, or indirectly via co-cultured non-neuronal cells (e.g., astrocytes), which release other factors that influence neurons directly. The reduced conditions provided *in vitro* have the advantage that direct effects on developing neurons can be tested, but the specific features of the cue conditions subjected to *in vivo* (i.e., concentration, localization, co-factors, mechanical environment, three-dimensional) may result in very different responses of developing neurons *in vivo. In vitro* findings inform and support work performed *in vivo*, and we will highlight here where there are discrepancies or if supporting *in vivo* evidence is lacking.

Acute bath application of growth factors can produce sudden and dramatic outcomes on neurite outgrowth, which can be useful for assessing receptor activity and rapid downstream signaling events. However, any effects on growth cone morphology or motility must be interpreted with caution, as growth cones will never encounter a cue in this manner *in vivo*. Growth factors are most commonly secreted or released from a tissue source and may become immobilized within the ECM or upon other cells (Billings and Pacifici, 2015; Balasubramanian and Zhang, 2016), which often leads to functional concentration gradients. Therefore, increasingly sophisticated *in vitro* assays are being developed in an attempt to more accurately reflect *in vivo* conditions. Over the last several decades, investigators have developed methods to challenge growth cones with local sources of cues, such as by binding cues to beads or releasing cues from a micropipette, or by positioning neurons near neighboring cells or within microfluidic chambers (Pujic et al., 2008). Now even more complex methods are being used, such as immobilized gradients of growth factors, gradients bound to elastic substrata, diffusion gradients within three-dimensional matrices and multi-cue conditions, with the goal to more accurately recapitulate *in vivo* conditions.

**FIGURE 1:**
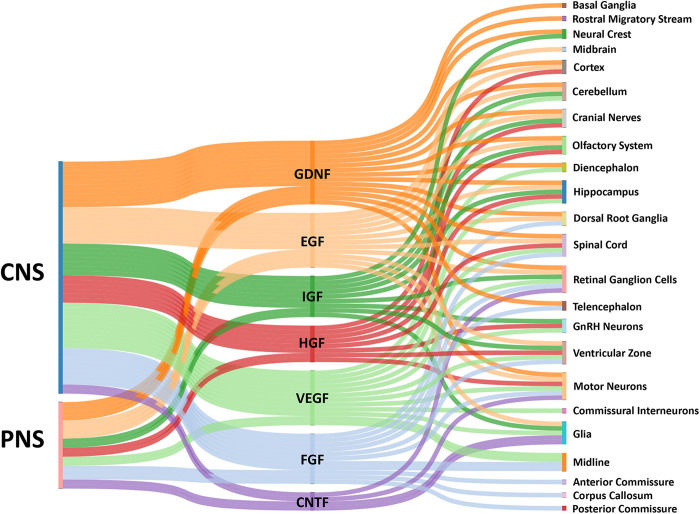
Sankey diagram illustrating influence of growth factors across the CNS and PNS. The colored bars indicate where expression of a growth factor has been identified in either the CNS or PNS *in vivo*. Multiple bars from a single growth factor to a single target implies input from both the CNS and PNS. The localization of growth factors in particular combinations provides complex influences on growth cones for the assembly of the nervous systems by providing temporal-spatial cues for axon guidance.

In this review, we discuss a number growth factors that have clear roles in axon guidance (Figure 1). These growth factors include ciliary neurotrophic factor (CNTF), epidermal growth factor (EGF), fibroblast growth factor (FGF), glial cell line-derived neurotrophic factor (GDNF), hepatocyte growth factor (HGF), insulin-like growth factor (IGF), and vascular endothelial growth factor (VEGF). While many of these growth factors can indirectly influence network assembly by regulating the expression of traditional axon guidance cues, here we focus on how they directly influence neuronal morphogenesis. Note that the neurotrophins (nerve growth factor, brain derived neurotrophic factor, neurotrophin-3, and neurotrophin-4/5), which are known to have important roles in axon guidance, will not be discussed as they have been reviewed previously (Guthrie, 2007; Lykissas et al., 2007). After presenting ligand family and cognate receptor distributions, we focus on studies that have examined the direct effects of these growth factors on axon extension of cultured primary neurons (Table 1). However, it is important to note that effects of growth factors on neuronal morphogenesis likely depend on culture conditions and any observed effects do not prove these factors operate in this manner *in vivo*. However, reduced conditions are necessary to identify signaling mechanisms used by these factors. Evidence shows that these factors alter growth cone morphology and neurite extension through pathways that signal through cytoskeletal, as well as transcriptional machinery.

**TABLE 1:** Growth factors have a wide variety of effects on the morphogenesis of developing neurons.

Growth factor	Receptor	*In vitro* guidance	*In vitro* extension	Citations
CNTF	CNTFRα	ND	Axon extension, arborization	Stahl and Yancopoulos, 1994; Syed et al., 1996; Oyesiku and Wigston, 1996; Selvaraj et al., 2012; Askvig and Watt, 2015
EGF	ND	ND	Axon extension	Morrison et al., 1987; Rosenberg and Noble, 1989; Kornblum et al., 1990; Tsai et al., 2010
Nrg-1	ErbB2 ErbB4	ND	Axon extension/branching, spines	Bermingham-McDonogh et al., 1996; Gerecke et al., 2004; Cahill et al., 2013; Modol-Caballero et al., 2017; Rahman-Enyart et al., 2020
Nrg-2	ErbB3	ND	Axon extension	Nakano et al., 2016; Rahman-Enyart et al., 2020
Nrg-3	ErbB4	ND	Axon extension/inhibition	Rahman-Enyart et al., 2020
Nrg-4	ErbB4	ND	Dendrite elaboration	Paramo et al., 2018
FGF2	FGFR1 and 3 Cell adhesion molecules	Chemoattraction/repulsion	Axon extension/inhibition, filopodia initiation, branching	Walicke et al., 1986; Williams et al., 1994; McFarlane et al., 1995, 1996; Szebenyi et al., 2001; Webber et al., 2003; Shirasaki et al., 2006; Boscher and Mege, 2008; Csanaky et al., 2019
FGF4	FGFR1	ND	Axon extension	Shirasaki et al., 2006
FGF8	FGFR1	Chemoattraction	Axon extension	Shirasaki et al., 2006
FGF9	FGFR1	ND	Axon extension	Shirasaki et al., 2006
GDNF	Ret	Chemoattraction	Axon extension	Lin et al., 1993; Schafer and Mestres, 1999; Dudanova et al., 2010; Miwa et al., 2013
GDNF	NCAM/GFRα1/Ret	Chemoattraction/Repulsion(confers)	Dendrite elaboration, branching, spine development	Kramer et al., 2006; Charoy et al., 2012; Irala et al., 2016; Bonafina et al., 2019
HGF	c-Met	Chemoattraction	Axon extension, branching, dendritic outgrowth	Ebens et al., 1996; Maina et al., 1997, 1998; Caton et al., 2000; Gutierrez et al., 2004; Sanford et al., 2008
IGF	IGF1R	Chemoattraction	Axon extension, branching	Recio-Pinto et al., 1986; Jones et al., 2003; Salie and Steeves, 2005; Ozdinler and Macklis, 2006; Sanford et al., 2008; Scolnick et al., 2008; Xiang et al., 2011
VEGF	VEGFR2 (Flk1)	Chemoattraction	Axon extension, branching	Sondell et al., 1999; Ruiz de Almodovar et al., 2011; Luck et al., 2019
VEGF164	Nrp1	Chemoattraction	Axon extension	Erskine et al., 2011

## Growth Factors and Receptor Tyrosine Kinases (RTK) Are Expressed Widely in the Developing Nervous System

### Ciliary Neurotrophic Factor (CNTF)

Ciliary neurotrophic factor was originally isolated from chick intraocular tissue where it was identified as a neurotrophic factor for its cell survival effects on the ciliary ganglion neurons (Adler et al., 1979). CNTF binds the CNTF receptor alpha (CNTFRα) subunit, which is a GPI-anchored ligand-binding subunit that interacts with glycoprotein 130 (gp130) and leukemia inhibitory factor receptor beta (LIFRβ) to form a functional transmembrane signaling complex (Ip N. Y. et al., 1992; Irala et al., 2016; Davis et al., 1993). Since then the modulatory effects of CNTF have been explored in a range of cell types from motor neurons (MNs) to oligodendrocytes (Sendtner et al., 1994; Talbott et al., 2007) and related diseases (Miller et al., 1996).

In vertebrates, mice lacking CNTF appear largely normal through adulthood (with only modest increased neuronal death as they age), while those lacking CNTFRα die perinatally (DeChiara T. Met al., 1995), suggesting CNTFRα may have additional ligands. Yet CNTF is widely expressed in glial cells across both the central and peripheral nervous systems, both during development and adulthood (Stockli et al., 1991; Sleeman et al., 2000). Particularly enriched in the sciatic and optic nerves, Schwann cell-specific expression is thought to be critical for the long-term survival and maintenance of these critical nerves. In support for roles in development, the CNTFRα receptor complex was detected in the ventral MNs of the spinal cord as early as E11.5 by immunohistochemistry (Gregg and Weiss, 2005), during periods of active axon pathfinding. Both CNTFRα and co-receptor LIFRß were also detected in the lateral geniculate along the ventricle, increasing substantially from embryonic day 9.5-11.5 (E9.5-11.5) (Gregg and Weiss, 2005).

Ciliary neurotrophic factor signaling has also been widely explored in the adult retina, particularly with regards to the maintenance of photoreceptor survival and the retinal pigment epithelium (Harada et al., 2002; Li et al., 2018). During development, CNTF expression in the retina rises steadily with age from E15.5-adulthood, with a similar pattern detected by RT-PCR for CNTFRα (Kirsch et al., 1997). CNTFRα was later detected in approximately one-third of developing Müller glia, but not photoreceptors, by laser capture and RT-PCR (Wahlin et al., 2004). While CNTFRα expression is also clearly detected in retinal ganglion cells (RGCs) by P0 (Kirsch et al., 1997) and is maintained throughout adulthood (Beltran et al., 2003), we will not discuss the extensive literature pertaining the effects of CNTF on optic nerve regeneration (Li H. J. et al., 2017).

### EGF/Neuregulins/ErbB Family

The Epidermal Growth Factor (EGF) family of receptors includes four receptors: EGFR (aka ErbB1 or HER1), ErbB2, ErbB3, and ErbB4 (Harris et al., 2003). These RTKs can either homo or heterodimerize with the exception of ErbB2, which must form a heterodimer with one of the other three receptors (Harris et al., 2003). In addition to EGF, which has the highest affinity for EGFR, this family includes the neuregulin (Nrg) ligands 1–4, of which there are up to six isoforms of Nrg-1, the first three of which are most well studied, and we will discuss here.

At all developmental stages, EGF receptors appear to be highly expressed in neural progenitors along the sub-ventricular zone (SVZ) (Aguirre et al., 2010), supporting their role in maintenance of these stem cell niches and life-long neurogenesis. The early expression of these receptors and their ligands in several other key locations suggests a role of EGF in circuit development. Whole mount *in situ* hybridization of mouse embryos showed early expression of type I Nrg-1 along the dorsal column of the developing mouse spinal cord, while type III Nrg-1 expression is enriched in the MNs of the ventral column of the spinal cord, in DRGs, and several cranial nerves (vagal, trigeminal, and glossopharyngeal) as early as E9.5 (Meyer et al., 1997). Similarly, Nrg-1 isoforms are also expressed in the developing *Xenopus* spinal cord, myotome, branchial arches, and the eyes (Yang et al., 1999). More detailed expression patterns of rodent spinal cord cross sections showed ErbB4 in the dorsal and ventral spinal cord and skeletal muscle at E10 (Meyer et al., 1997). On the other hand, ErbB3 is enriched in DRGs, muscle, and developing Schwann cells, with little to no expression in the spinal cord. Beyond the spinal cord, the expression patterns in the perinatal brain showed expected high levels of expression in the ventricular zone and notable enrichment of ErbB4 in the olfactory epithelium, and cortical plate, while type III Nrg-1 was expressed in the mesencephalon, olfactory epithelium, and the dentate gyrus of the hippocampus (Meyer et al., 1997). Other studies have observed distinct ErbB receptor expression patterns in the developing mouse midbrain (Lopez-Bendito et al., 2006; Abe et al., 2009) and Nrg-1 expression in the developing DRG (Hancock et al., 2011). In postnatal rats, both receptor and ligand expression have been detected in the developing olfactory epithelium (Anton et al., 2004), as well as the cortex, hippocampus, and Purkinje cell layer of the cerebellum (Kornblum et al., 2000).

### Fibroblast Growth Factor Family

Fibroblast growth factor (FGF), was first isolated from cow brain and pituitary in the early 1970s and named for its mitogenic effects on fibroblasts (Armelin, 1973). The FGFs and FGF receptors (FGFRs) have since been widely studied in a variety of species and regions of developing nervous systems. The FGF family is made up of up to 22 ligands with four associated receptors FGFR1-4. While several of these ligands contain remarkable sequence homology (FGF11-14), they are classified as FGF homology factors, as they do not appear to bind FGF receptors (Itoh and Ornitz, 2004; Guillemot and Zimmer, 2011).

In vertebrates, FGF receptors and ligands are expressed broadly throughout development, indicating roles in early patterning and likely in axon pathfinding. FGFR1 and FGFR2 are expressed in the ventricular zone of the ventral forebrain and primordial lateral geniculate as early as E9.5 in the developing mouse (Vaccarino et al., 1999), suggesting a role for proliferation and differentiation of these progenitor pools. FGFR1 and FGF8 are also strongly expressed along the anterior telencephalic midline in mice (Smith et al., 2006) and similar midline expression of FGF8 is observed in developing zebrafish (Shanmugalingam et al., 2000). FGF2 is expressed at the midline during anterior and posterior optic commissure development in *Xenopus laevis* (McFarlane et al., 1995), suggesting roles in midline organization or commissural formation. This midline pattern is consistent with a variety of other species including grasshopper (Condron, 1999). Several other FGF ligands are expressed in the developing brain, with FGF-1, FGF-3, FGF-5, FGF-6, FGF-7, and FGF-8 showing temporally restricted patterns and FGF-6 in particular decreasing expression in the brain sharply after birth (Ozawa et al., 1996).

Fibroblast growth factors in the spinal cord also exhibit temporally defined expression patterns in progenitors, MNs, and dorsal root ganglion (DRG). FGFR1 is exclusively expressed in the ventro-lateral developing MNs (Shirasaki et al., 2006), while FGFR2 and FGFR3 expression is restricted to midline progenitor pools, but no FGFR4 expression was observed between E10.5 and 12.5 in mice. On the other hand, four FGFRs were detected specifically within subsets of developing MNs by *in situ* hybridization, as well as FGFR2 in the developing DRG at E10.5 (Kato et al., 1992). Perhaps the most compelling pattern for axon guidance is that of FGFRs in *Xenopus* RGCs, and FGF2 expression in the developing neuropil of the diencephalon where RGCs pass through immediately prior to innervating the optic tectum (McFarlane et al., 1995).

### Glial Cell Line-Derived Neurotrophic Factor

Glial cell line-derived neurotrophic factor (GDNF) was first isolated from a rat astrocytic cell line (B49) and characterized for its potent trophic effects on dopaminergic neurons from the substantia nigra (Lin et al., 1993). Since its discovery, GDNF expression and function has been widely studied across the nervous system (Cortes et al., 2017). Three other structurally related members were identified from the GDNF family: neurturin, persephin, and artemin, which bind preferentially to GFRα 2-4 respectively, with GFRα1 preferentially binding to GDNF (Airaksinen et al., 2006). GFRα receptors are GPI-anchored so signaling requires co-receptors, which are typically transmembrane Ret RTKs, which can either be in *cis* on the same cell, or in *trans* with adjacent cells. Importantly, Ret-independent signals downstream of GDNF are also possible, which involve GFRα1-associated NCAM heterodimers (Paratcha et al., 2003), as discussed below.

As expected, given its effects on dopaminergic neurons, GDNF is clearly detected by *in situ* hybridization in the developing striatum and caudate putamen (Stromberg et al., 1993). Post-natal day five (P5) rats express both GDNF mRNA and protein around dopaminergic cells in the substantia nigra, with these enriched “patches” dissipating to diffuse ubiquitous staining by P14 (Oo et al., 2005). Outside the striatum, GDNF mRNA was detected by RT-PCR throughout the rat CNS, with particular enrichment in the spinal cord and striatum around birth, though lower levels were also detected in the cerebellum, diencephalon, and telencephalon (Choi-Lundberg and Bohn, 1995). GDNF expression was also observed in a subset of developing muscle cells (Moore et al., 1996). The C-Ret receptor is expressed in developing neural crest, cranial ganglia, and later in the developing eye of mouse embryos by whole mount *in situ* (Pachnis et al., 1993). Other work identified GFRα1 receptor expression in the embryonic olfactory bulb (Zechel et al., 2018) and in developing mouse DRGs along with co-receptor Ret (Chen et al., 2017). Interestingly Ret is also expressed by chemosensory geniculate neurons (Donnelly et al., 2018) and by the trigeminal axons that project to the dental pulp, which in turn expresses GDNF (Donnelly et al., 2019).

In the developing mouse cortex, GDNF and its receptor GFRα1 are highly expressed from E13.5–15.5 with GFRα1 expression enriched in Tbr1 positive neurons in the cortical plate (Bonafina et al., 2018). In the spinal cord, GDNF is expressed specifically in the floorplate of the developing spinal cord prior to midline interneuron crossing (Charoy et al., 2012). In addition, GDNF is expressed in the dorsal limb mesenchyme, where it serves as a chemoattractant for lateral motor column (LMC) MNs (Kramer et al., 2006).

In the adult, GDNF is detected by immunohistochemistry in the human cortex, cerebellum, hippocampus, and occipital lobe (Kawamoto et al., 2000), and in rodent thalamus, hippocampus, olfactory bulb, motor nuclei of cranial nerves, and deep cerebellar nuclei (Trupp et al., 1997). Of particular relevance for ongoing circuit development and plasticity throughout life, GFRα1 expression is especially enriched in the dentate gyrus and specifically within newly born and differentiated neurons in adult rodents (Irala et al., 2016; Bonafina et al., 2019).

### Hepatocyte Growth Factor (HGF)

Hepatocyte growth factor, also known as scatter factor (Naldini et al., 1991), is a soluble protein found to promote hepatocyte growth and liver generation (Nakamura et al., 1986), as well as promote the dissociation of epithelial cells in culture (epithelial to mesenchymal transition) (Stoker and Perryman, 1985). HGF signals through c-Met, a RTK, also referred to as tyrosine-protein kinase Met and hepatocyte growth factor receptor (HGFR) (Baldanzi and Graziani, 2014).

Although HGF is expressed and secreted by mesenchymal cells throughout the organism with many roles in cell survival, migration, cancer metastasis, and wound healing, there is also clear evidence across numerous species for roles in neural development. In *C. elegans*, HVS-1 is a HGF homolog expressed in the chemosensing nociceptive ADL neurons (Li C. et al., 2012). In the developing mammalian brain, c-Met is detected in post-mitotic neurons in the cortical plate, as well as in the intermediate zone of the developing cortex (Achim et al., 1997). c-Met expression is also elevated in specific populations of developing projection neurons within the neocortex and limbic system, including the hippocampus, with enrichment of c-Met immunoreactivity in axons during periods of neurite extension and synaptogenesis (Judson et al., 2009), consistent with a role in synaptic maturation (Qiu et al., 2014). HGF expression in turn is elevated in differentiating neurons but appears overall to be most prominently detected in the embryonic subventricular and ventricular zones (Jung et al., 1994), suggesting a role in migration and differentiation of neurons in the cerebral cortex. Evidence for a role for HGF in neuronal migration also comes from its expression in the nasal cavity and along axons of the developing olfactory epithelium. HGF is expressed in an increasing gradient toward the border between the nose and telencephalon in the embryonic nasal mesenchyme (Thewke and Seeds, 1996). Moreover, the c-Met receptor, as well as tissue plasminogen activator (tPA), the catalytic activator of Pro-HGF (see below), are both highly expressed in GnRH neurons exclusively during the period in which these cells migrate from the olfactory epithelium into the forebrain (Thewke and Seeds, 1996; Giacobini et al., 2007).

Perhaps the most compelling evidence for a role of HGF signaling in axon guidance comes from developing MNs of spinal cord and brainstem. Within the limb buds, HGF expression has been localized to specific MN targets in the myotome, as well as branchial and pharyngeal arches (Ebens et al., 1996; Caton et al., 2000; Isabella et al., 2020). c-Met in turn is expressed by spinal MNs and cranial motor nerves (Ebens et al., 1996; Caton et al., 2000; Isabella et al., 2020).

### Insulin-Like Growth Factor (IGF)

The insulin-like growth factor family is made up of two ligands (IGF-1 and IGF-2) and two cell surface receptors (IGF1R and IGF2R), although no intrinsic tyrosine kinase or other enzymatic activity has been reported for IGF2R (O’Kusky and Ye, 2012). In addition, IGF1R functions as a co-receptor for the insulin receptor (InR) (Moxham et al., 1989).

Insulin-like growth factor signaling appears to be evolutionarily conserved from *C. elegans* to *Drosophila* to rodents (Garcia-Segura et al., 1991; Kenyon et al., 1993; Nassel and Vanden Broeck, 2016) with a significant regulatory role for body and brain size, feeding behavior, metabolism, fecundity, and lifespan (Wrigley et al., 2017). Loss of IGF-1 results in a robust reduction in white matter and oligodendrocytes throughout the brain and spinal cord (Beck et al., 1995). Overall, IGF-1 expression appears to decline with age, showing much less expression in the adult rat brain compared to early neonatal animals, which show robust immunoreactivity by embryonic neurons, trigeminal ganglia, and astrocytes (Garcia-Segura et al., 1991). In contrast, IGF1R expression in the brain remains relatively high throughout adulthood, particularly in the neurogenic regions of the adult brain, hippocampus, SVZ, and olfactory bulbs (Nieto-Estevez et al., 2016).

Examining more specific neural networks and brain regions, IGF-1 is expressed by gonadotropin releasing hormone (GnRH) neurons in salmon and zebrafish, suggesting a role for IGF signaling in reproductive signaling axis development (Ando et al., 2006; Onuma et al., 2011). Consistent with regulation of neuronal migration, IGF1R is expressed specifically at the tips of growing GnRH neurons of the arcuate nucleus in the hypothalamus (Decourtye et al., 2017). Sustained expression of both receptor and ligand has also been observed in the hippocampus and appears to play a role in learning and synaptic reorganization (Trejo et al., 2007). In the chick, IGF-1 may regulate the migration of neural crest cells as IGF-1 is expressed in the apical ectodermal ridge of the wing bud (Schofer et al., 2001), while expression of IGF-1 in the olfactory bulbs indicates a role in the rostral migration streams (Hurtado-Chong et al., 2009). IGF-1 is also expressed in young (P10) cerebellum of mice where it is regulated by circadian cycles with increased levels detected during light periods (Li Y. et al., 2012).

In the developing E16.5 mouse retina, IGF-1 is expressed in specific RGCs that will project to the contralateral LGN, while high affinity IGF binding protein-5 (IGFBP-5) mRNA is detected in RGCs that project ipsilaterally (Wang et al., 2016). While the authors did not explore the downstream targeting, the timing of this differential gene expression during decussation suggests some role in guidance to the correct LGN targets.

### Vascular Endothelial Growth Factor (VEGF)

Vascular endothelial growth factor (VEGF) was first isolated from solid tumors and was named tumor-angiogenesis factor (Folkman et al., 1971). It has now become clear that VEGF is involved in blood vessel development during all stages of life (Apte et al., 2019) and that loss of a single copy of VEGF leads to embryonic lethality in rodents (Ferrara et al., 1996). While vascularization has been the primary focus around much VEGF research, it is clear VEGF has broader functions in development. For example, evolutionary analysis has identified highly homologous VEGF ligands and receptors in invertebrates which have no vasculature. Here other roles have been shown, such as regulation of non-endothelial cell migration, neuritogenesis, and the development of branching organs like the trachea (Kipryushina et al., 2015).

It is now understood that VEGF contains five family members (A-E) (Apte et al., 2019), with the most extensively studied and relevant for this review being VEGF-A, which we will continue to refer to here simply as VEGF. Alternate splicing of VEGF leads to multiple isoforms in humans consisting of 121, 165, or 189 amino acids, along with less common isoforms 145 and 183 (Apte et al., 2019). The ability of these different isoforms to bind heparin through heparin-binding domains with distinct affinities is a key feature distinguishing isoform, with VEGF121 being the most diffusible, and VEGF189 most highly bound to the ECM (Apte et al., 2019). All isoforms of VEGF bind to the receptors VEGFR-1 (Flt1) and VEGFR-2 (Flk1/FDR). The two common longer isoforms of VEGF also have a high affinity for neuropilin 1 (Nrp1) (Tillo et al., 2015).

Due to its essential role in vascular development throughout the animal, VEGF and its RTKs are expressed in a spatio-temporal manner consistent with organizing proper ingression of vessels (Eichmann and Thomas, 2013). VEGF is synthesized and released in many locations, including the ventricular neuroectoderm and the midline of the developing neural tube (James et al., 2009). More compelling for potential axon guidance is the expression of VEGF in the floorplate, ventral midline, and motor columns of the developing spinal cord while its receptor, Flk1, is expressed by commissural interneurons prior to crossing (Ruiz de Almodovar et al., 2011).

In the mouse cerebellum, matrix-binding VEGF164 is expressed in the cell bodies and on the dendrites of Purkinje neurons while the granule cells that will eventually synapse onto these dendrites express Flk1 receptors (Ruiz de Almodovar et al., 2010). Similarly, migrating GnRH neurons born in the olfactory epithelium also express VEGF receptors Nrp1 and Flk1 (Cariboni et al., 2011). Developing pyramidal neurons of the hippocampus, but not interneurons in CA3, also express VEGF receptor Flk1, while VEGF is expressed by several cell types including pyramidal neurons and GFAP positive astrocytes (Harde et al., 2019; Luck et al., 2019). VEGF is also expressed in the portions of the diencephalon that will become the primary substrate for optic chiasm development, while VEGF receptor Nrp1 is highly expressed in the RGCs that cross the midline (Erskine et al., 2011).

### Ligand Secretion or Proteolytic Release of Growth Factors

Most growth factors are synthesized as premature, inactive pre-pro-proteins which must be processed into their biologically active forms by cleaving the signal peptide and pro-domain either in the secretory pathway or extracellularly. Pro-domains are thought to assist in folding and stabilization of the mature domain, and to direct intra- and extracellular localization, storage, and bioavailability. This added layer of regulation combined with local expression patterns provides a powerful means to control ligand availability and local concentration, especially as some released active ectodomains are diffusible while others become tethered to cell membranes or the ECM, such as to heparin sulfate proteoglycans.

For those growth factors secreted as pro-forms and activated extracellularly, there are several classes of proteolytic enzymes known to regulate ligand availability in this way. For example, β-Secretase (β-site amyloid pre-cursor protein cleaving enzyme 1, BACE1), plasminogen activators, zinc-dependent matrix metalloproteases (MMPs), and a disintegrin and metalloprotease domain-containing enzyme (ADAMs, aka α-secretase) family members are responsible for the partial proteolysis and activation of several growth factors (Page-McCaw et al., 2007; De Strooper et al., 2010).

One of the most well-studied and best examples of growth factors that are proteolytically activated extracellularly are the EGF and Nrg family of ligands. All EGF/Nrg isoforms are synthesized as single- or dual-pass transmembrane proteins and require proteolytic cleavage to either release soluble, receptor-binding ectodomains or act through juxtacrine signaling (Sahin et al., 2004; Czarnek and Bereta, 2020). Interestingly, specific stimuli such as NMDA receptor signaling or PKC activation are known to signal through particular ADAM metalloproteases to activate EGF ligands (Dang et al., 2011; Vullhorst et al., 2017). Complicating signaling further are findings that reverse signaling from ErbB receptors to Nrg-1 ligands can activate gamma secretase-dependent proteolytic release and nuclear translocation of the intracellular domain of type III Nrg1 (Bao et al., 2004). The intracellular domain of Nrg1 is involved in the patterning of cortical dendrites (Chen et al., 2010), as well as guidance of DRG axons centrally and into the periphery (Hancock et al., 2011). Interestingly, the intracellular domain of Nrg1 may signal locally within growth cones to regulate the surface expression of Nrp1 to control the sensitivity of growth cones to Semaphorin3A (Sema3A) (Hancock et al., 2011).

ECM proteins, such as proteoglycans, often have a high affinity for secreted growth factors and provide another means for regulating growth factor signaling through local immobilization. For example, many growth factors bind heparin sulfate proteoglycans, including FGFs, HGF, and IGF (Billings and Pacifici, 2015; Zhang et al., 2019). IGF is similarly localized by seven high-affinity IGF-binding proteins (along with several other low-affinity IGFBPs), which require proteolytic cleavage to release IGF to bind to its receptor for local signaling (Allard and Duan, 2018). Pregnancy-associated plasma protein-aa (pappaa), is one such metalloprotease known to cleave IGF-binding proteins to release IGF-1 (Oxvig, 2015). Mutations in pappaa and disruptions in proteolytic cleavage of IGF binding proteins have been shown to impact development of the retina (Miller et al., 2018), as well as the acoustic startle habituation learning in larval zebrafish (Wolman et al., 2015).

## Growth Factors Modulate Axon Outgrowth and Guidance *in vitro*

### Ciliary Neurotrophic Factor

A number of studies show that CNTF promotes neuronal survival, axon formation and arborization, as well as neurite regeneration for several classes of neurons across different species *in vitro*. Early studies showed that CNTF promoted neurite outgrowth of acoustic and spiral ganglion neurons in a dose-dependent manner, which was further enhanced by BDNF (Hartnick et al., 1996; Schwieger et al., 2015). Interestingly, the outgrowth promoting effects of CNTF, both with and without BDNF, were abolished at higher CNTF concentrations (Hartnick et al., 1996), but the mechanisms for this effect were not explored. CNTF also promotes axon extension by chick spinal MNs and interneurons, but unlike acoustic ganglion neurons, the dose-dependent effect of CNTF plateaus at higher concentrations (Oyesiku and Wigston, 1996). More recent work within organotypic hypothalamic slice culture showed that CNTF stimulated the arborization of oxytocin containing neurons, but these effects may be indirect through CNTF activation of astrocytes (Askvig and Watt, 2015). The growth-promoting effects of CNTF extend phylogenetically back to invertebrates, such as interneurons from the mollusk *Lymnaea*. Compared to NGF treatment, which induced both outgrowth and synapse formation by *Lymnaea* interneurons, CNTF only supported neurite extension (Syed et al., 1996). These data suggest that CNTF regulates neuritogenesis and regeneration, but not later phases of neural development, such as synaptogenesis. Moreover, we can find no evidence that CNTF is able to guide neurons using assays performed *in vitro*, such as gradient turning assays. Since CNTF and its receptors are expressed in patterns that suggest it may function in axon guidance, future experiments should address this possibility *in vitro*.

### EGF and Neuregulins

Epidermal growth factor is the most well-studied growth factor discussed in this review (Dolgin, 2017), as it influences many cellular functions, including cell motility and cancer metastasis (Lindsey and Langhans, 2015; Vullhorst et al., 2017). Although fewer studies have examined effects on developing neurons, it is clear that EGF, and structurally similar Neuregulins 1–4, can directly and indirectly influence neurite extension. Early studies showed that chronic EGF treatment promotes neurite extension from several classes of primary neurons (Morrison et al., 1987; Rosenberg and Noble, 1989; Kornblum et al., 1990). Subsequent studies identified some underlying mechanisms of chronic EGF-induced neurite extension in mouse cortical neurons, as well as rat DRG neurons (Tsai et al., 2010). Nrg treatment supports neuronal survival and neurite outgrowth by spinal MNs, DRGs, RGCs, hippocampal and cortical neurons as well (Bermingham-McDonogh et al., 1996; Gerecke et al., 2004; Nakano et al., 2016; Modol-Caballero et al., 2017; Rahman-Enyart et al., 2020). Nrgs have also been shown to enhance dendrites and dendritic spine formation by cortical neurons (Cahill et al., 2013; Paramo et al., 2018). However, most studies performed to date have only tested long-term effects of EGF and Nrgs, which signal through transcription-dependent pathways that regulate neuronal differentiation and survival. It will be interesting and important to determine whether EGF and Nrgs also have rapid and local effects on growth cone motility, as this is undoubtedly the case for many motile non-neuronal cells (Keller et al., 2017).

### Fibroblast Growth Factor

FGF2 has concentration, context, and neuronal class-dependent effects on axon extension, branching, and guidance. For example, a pioneering study demonstrated that FGF2 (aka bFGF) enhanced neurite outgrowth of rat hippocampal neurons when bound to a heparin substratum, but in solution had no effect on axon extension of neurons growing upon laminin (Walicke et al., 1986). On the other hand, chronic FGF2 treatment promotes neurite extension by *Xenopus* RGCs growing on poly-ornithine/laminin (McFarlane et al., 1995), which may be due to differences in species, neuronal class, or culture conditions. While chronic stimulation with FGF2 could work through transcriptional changes, acute treatment with soluble FGF2 also promoted rapid, FGF receptor-dependent acceleration of RGC axon extension (McFarlane et al., 1996), suggesting local effects on growth cone motility. Oddly, while global treatment with FGF2 stimulates RGC extension, local application to RGC growth cones repels axon outgrowth (Webber et al., 2003). However, FGF produced by the dermomyotome selectively attracts axons of medial-class spinal MNs *in vitro* (Shirasaki et al., 2006). In this study, several different FGF family members were found to promote MN axon extension (FGF2, FGF4, FGF8, FGF9). In contrast to these findings, other groups found that FGF2 either had no effect on axon extension or even slowed terminal extension but promoted robust axonal branching (Aoyagi et al., 1994; Szebenyi et al., 2001). In cortical pyramidal neurons, acute FGF2 treatment or local application of FGF2 coated beads induced rapid sprouting of new filopodia and axonal branching (Szebenyi et al., 2001). It is interesting to note that FGF receptors can also be activated directly by cell adhesion molecules (CAMs) such as L1, NCAM, and cadherins to promote axon outgrowth and neurons extending upon cells expressing these CAMs are acutely inhibited by soluble FGF2 (Williams et al., 1994; Boscher and Mege, 2008). The complex effects of FGF2 on neurons *in vitro* make it clear that FGF2 likely has diverse and context-dependent influences on developing neurons *in vivo* and may serve as a bi-functional axon guidance factor in a manner similar to many classic axon guidance cues.

### Glial Cell Line-Derived Neurotrophic Factor

Glial cell line-derived neurotrophic factor has been the focus of intense research in recent years, as this neurotrophic factor has clear roles in axon guidance of multiple classes of neurons. Pioneering work identified GDNF as a trophic factor for midbrain dopaminergic neurons and showed that it enhanced process extension *in vitro* (Lin et al., 1993). Subsequently, GDNF was shown to specifically promote neurite elongation in dissociated myenteric plexus neurons in a dose dependent manner, while having no effect on glial or non-neuronal cell morphology (Schafer and Mestres, 1999). Chemotropic activity of GDNF was later identified toward several classes of neurons (Paratcha et al., 2006; Paratcha and Ledda, 2008; Schuster et al., 2010; Miwa et al., 2013). However, perhaps the most well characterized role of GDNF as a chemoattractant *in vitro* comes from mouse LMC MNs. Analysis *in vitro* shows that GDNF stimulates axon extension from both medial and lateral LMC MNs, but only serves as an attractant to lateral LMC MNs when tested in a Dunn chamber (Dudanova et al., 2010). In an attempt to model conditions *in vivo*, counter gradients of EphrinA5 (repulsive force) and GDNF (attractive force) produced more robust turning responses than individual cues, suggesting MN growth cones integrate these signals. This study found that GDNF also reduced the inhibitory effects of EphrinAs, and this effect depended on functional Ret receptors. Adding to the diverse functions of Ret receptors in MN axon guidance, EphrinA receptors on lateral LMC MNs function in reverse signaling with Ret receptors to promote growth toward EphA ligands in the dorsal limb (Bonanomi et al., 2012). Therefore, the Ret RTK acts as a multi-functional coreceptor with EphrinA and GFRα1 to promote outgrowth downstream of EphrinA and GDNF, respectively. Alternatively, GDNF can signal through NCAM/GFRα1 receptor complexes (Paratcha et al., 2006; Paratcha and Ledda, 2008), which are involved in midline crossing by commissural interneurons (CIs) in the spinal cord (Charoy et al., 2012). Here, GDNF at the midline activates repulsion from Sema3B through NCAM/GFRα1 receptors (Charoy et al., 2012). The NCAM/GFRα1 receptor complex is necessary for proper hippocampal dendritic outgrowth, branching and spine development downstream of GDNF as well (Irala et al., 2016).

### Hepatocyte Growth Factor

Hepatocyte growth factor is secreted from limb mesenchyme and was first identified as a neurotrophic growth factor toward rat spinal MN axons (Ebens et al., 1996). Interestingly, the neurotrophic activity on MNs appeared to be specific to HGF, as several different growth factors tested were not able to promote MN axon outgrowth into collagen gel, including GDNF, FGF2, EGF, and CNTF. However, these results may be highly context dependent, as we now know that GDNF strongly promotes axon extension by lateral LMC MNs (described above). Subsequently, these findings were confirmed using cranial MNs, which were found to be strongly attracted toward branchial arch mesenchyme and HGF beads in collagen gel assays (Caton et al., 2000). In addition to its effects on axon outgrowth, exogenous application of HGF has been shown to promote dendrite extension and branching by layer 2 pyramidal neurons in culture (Gutierrez et al., 2004). Further, treatment of pyramidal neurons with function-blocking antibodies to HGF suggests that HGF released from neurons has paracrine effects on dendritogenesis (Gutierrez et al., 2004).

### Insulin-Like Growth Factor

Insulin-like growth factor has numerous roles during development, including regulating cell proliferation and survival, so loss of function mutations in either *Igf1, Igf2, or Igf1r* results in severe growth deficiencies (DeChiara et al., 1990; Liu et al., 1993). Similarly, IGF regulates neuronal proliferation and survival, but also has important roles in axon outgrowth and guidance. An early study showed that Insulin and IGF (with greater potency) promoted axon extension by chick sympathetic and sensory neurons (Recio-Pinto et al., 1986). Subsequent studies found that IGF-1 enhanced migration and branching of postnatal DRG neurons (Jones et al., 2003), as well as axon extension of embryonic DRG neurons (Sanford et al., 2008; Xiang et al., 2011). More recently, IGF was shown to play a specialized role in corticospinal motor neuron (CSMN) outgrowth *in vitro* (Ozdinler and Macklis, 2006). IGF-1 specifically stimulates axon extension by CSMNs without affecting secondary branching. The effect of IGF-1 sharply contrasted with BDNF, which robustly enhanced CSMN branching, but had no effect on axon length (Ozdinler and Macklis, 2006). Similar effects of IGF-1 were observed with vestibulospinal and spinal projection neurons from the raphe nucleus (Salie and Steeves, 2005). IGF-1 appears to act by stimulating growth cone motility, as local contact with IGF-1 coated beads results in rapid acceleration of CSMN axon outgrowth (Ozdinler and Macklis, 2006), suggesting IGF-1 is not functioning only as a survival factor. Moreover, a soluble gradient of IGF-1 serves as a chemoattractant for both olfactory sensory and cerebellar granule neuron growth cones (Scolnick et al., 2008), but not rat DRG neurons (Sanford et al., 2008). It is not clear why IGF-1 stimulates outgrowth, but not chemotropism of DRG axons. Mouse cortical neurons also exhibit chemotropic turning toward graded IGF-1 (and BDNF) within 3D collagen and matrigel, which appears to depend on matrix rigidity (Srinivasan et al., 2014). However, this study altered matrix rigidity by increasing collagen ligand concentration, which has confounding effects on ligand density (Nichol et al., 2019).

### Vascular Endothelial Growth Factor

It is clear from a number of *in vitro* studies over the last 10 years that VEGF can have rapid and diverse effects on the cytoskeleton to influence neuronal morphogenesis. Pioneering work showed that VEGF had dose-dependent neurotrophic effects on mouse superior cervical ganglion and DRG neurons (Sondell et al., 1999). More recently it was demonstrated using a Dunn chamber that mouse CI growth cones exposed to graded VEGF exhibit significant chemoattractive turning toward VEGF (Ruiz de Almodovar et al., 2011). Chemoattraction toward VEGF required VEGFR2 (Flk1), as receptor neutralizing anti-Flk1 antibodies abolished all growth cone turning (Ruiz de Almodovar et al., 2011). On the other hand, chronic treatment of young hippocampal neurons at 1 DIV with VEGF increased axon branch number and length, without affecting primary neurite lengths. Further, using live F-actin imaging of hippocampal pyramidal neurons, the authors found that acute VEGF treatment rapidly increased axon branch formation from existing F-actin patches (Luck et al., 2019). In cooperative work performed in hippocampal slice cultures, dendrite length, branching, and spine density of CA3 pyramidal neurons were reduced in VEGFR2 receptor KO neurons (Harde et al., 2019). Consistent with this, acute treatment of hippocampal neurons at 14 DIV with VEGF promotes rapid spine formation, which depended on VEGFR2 endocytosis (Harde et al., 2019). While VEGF does not appear to affect axon outgrowth by hippocampal neurons, it does promote axon outgrowth and increase growth cone size of DRG neurons, which requires both VEGFR2 and Nrp1 (Olbrich et al., 2013; Schlau et al., 2018). Interestingly, Sema3E stimulates axon extension by subiculum neurons through VEGFR2-Nrp1 co-receptors (Bellon et al., 2010), but is unable to promote chemotropic guidance toward Sema3E by CIs, which also express these receptors (Ruiz de Almodovar et al., 2011).

## Growth Factor Receptors Recruit Common Signaling Pathways

### Ciliary Neurotrophic Factor

Ciliary neurotrophic factor binds the CNTFRα subunit, leading to recruitment of other receptor subunits and activation of cytosolic tyrosine kinases (Jak/Tyk) (Stahl and Yancopoulos, 1994) and downstream transcriptional changes through phosphorylation of signal transducer and activator of transcription-3 (STAT3) (Selvaraj et al., 2012). These signals converge on pathways that regulate gene expression involved in neuronal survival and proliferation. Interestingly, STAT3 was recently shown to support neurite outgrowth of MNs by stabilizing the microtubule cytoskeleton through inhibition of stathmin, a microtubule destabilizing factor (Selvaraj et al., 2012). While these findings were demonstrated in progressive motor neuronopathy mutant MNs, similar activities may occur in normal developing neurons. In developing sensory neurons, CNTF acts through the non-canonical nuclear factor-κB (NF-κB) transcriptional system to promote neurite outgrowth as inhibition of NF-κB effectively abolished any increased process elongation due to CTNF stimulation (Gallagher et al., 2007).

### EGF and Neuregulins

While it is clear that EGF and Nrgs activate EGFR and ErbB receptors, other ligands signal through these receptors and EGF may modulate receptor activities in some conditions, complicating signaling and outcomes on cell motility. For example, a naturally occurring c-terminal fragment of the ECM protein versican promotes axon outgrowth through EGFR (Xiang et al., 2006). Moreover, similar to work described above regarding CAM-binding FGF receptors, EGF receptors bind CAMs such as L1 and NCAM in developing *Drosophila* (Islam et al., 2004). In a related study, neurite outgrowth by cerebellar granule neurons (CGNs) through homophilic NCAM interactions depends on inhibition of EGFR signaling (Povlsen et al., 2008), suggesting active EGFR inhibits axon outgrowth in this context. Similarly, the inhibitory effects of myelin inhibitors and chondroitin sulfate proteoglycans on axon regeneration by CGNs and DRG neurons can be blocked by preventing activation of EGFR (Koprivica et al., 2005). Therefore, the effects of EGFR activation appear to be highly context-dependent, with opposite effects on neurite extension observed under different conditions.

Importantly, little is known about the rapid and local effects that EGF or Nrgs may have on growth cone motility, axon guidance, or local branching. As it is well understood that EGF directly regulates the cytoskeleton to modulate motility of many non-neuronal cell types (Chang et al., 1995; Hazan and Norton, 1998), it is expected that EGF may regulate neurite extension by similar mechanisms. In non-neuronal cells, phospholipase C (PLC) is required for rapid EGF-induced local cofilin activation and barbed end actin formation, which are essential for initiation of nascent cell protrusions and turning toward a gradient of EGF (Mouneimne et al., 2004). EGF-dependent chemotaxis also requires global inactivation of cofilin by LIMK phosphorylation, which is believed to amplify local cofilin function and asymmetric actin polymerization (Mouneimne et al., 2006). As local actin polymerization is often the driving force behind growth cone turning (Gomez and Letourneau, 2014), it is likely that EGF influences signaling in neurons in a similar way. The Condeelis lab showed that EGF also generates a second peak of barbed actin through phosphoinositide-3 kinase (PI3K) activation to further promote actin polymerization at the leading edge of lamellipodia. Importantly, PI3K activation through EGF has been implicated in invadopodia formation, which are actin-rich basal protrusions that are associated with remodeling of the ECM and cancer metastasis (Eddy et al., 2017). Further investigation of EGF-dependent signaling in invadopodia formation shows that Src family kinases and downstream Abl-related non-RTK are required for EGF-induced cortactin phosphorylation, suggesting that an EGFR-Src-Arg-cortactin pathway mediates invadopodia formation and subsequent cell invasion (Mader et al., 2011). Therefore, EGF may play an essential role in invadopodia formation in developing neurons as well, as it has been shown that growth cones from different neuronal types and species generate protrusions structurally and functionally similar to invadopodia (Santiago-Medina et al., 2015; Wrighton, 2019). It is believed that growth cones use invadopodia to locally remodel the ECM to cross tissue barriers, such as MN exiting from, and DRG entry into the spinal cord from the periphery (Santiago-Medina et al., 2015; Nichols and Smith, 2019). Considering the extensive evidence for EGF as a determinant of cell motility and invasion, as well as its early expression in the developing nervous system, this growth factor likely has key roles in axon pathfinding.

### Fibroblast Growth Factor

Similar to other RTKs, binding FGF ligands lead to receptor dimerization and autophosphorylation of receptor kinase domains. Following recruitment of various adaptor proteins, several downstream signals that promote neurite outgrowth are activated in neurons, most prominently the Ras/extracellular signal-regulated kinase (ERK) and phosphatidylinositol-3 kinase (PI3K)/AKT pathways (Zhou and Snider, 2006). Importantly, upon ligand binding, receptor internalization is necessary for ERK1/2 activation (MacInnis and Campenot, 2002), signal termination by transport into late endosomes/multi-vesicular bodies, and eventual degradation in lysosomes (Platta and Stenmark, 2011). In addition to signaling in the cytosol, FGFRs translocate into the nucleus to regulate gene expression. To elucidate pathways that contribute to the regulation of axon outgrowth, optogenetics was used to control FGFR1 receptor activation on membranes, in the cytosol, and in the nucleus of PC12 cells (Csanaky et al., 2019). Here it was shown that light activation of only membrane bound FGFR1 resulted in ERK phosphorylation and increased neurite outgrowth. In contrast, neither activation of cytosolic nor nuclear FGFR1 in PC12 cells resulted in ERK activation or neurite outgrowth. Since the duration of receptor activation can have dramatic effects on functional outcomes, it is important to better understand mechanisms that regulate trafficking of FGFRs between distinct cellular locations.

### Glial Cell Line-Derived Neurotrophic Factor

Signaling downstream of GDNF is complex and poorly understood in growth cones, especially considering all the possible co-receptor combinations that have been identified. As GDNF signals that regulate transcription to influence cell survival have previously been described (Peterziel et al., 2002), here we focus on local signaling effects on growth cone motility. Canonical signaling involves GDNF binding to high affinity GFRα receptors and signal transduction through Ret RTKs. As GDNF can cause fast growth cone turning responses (Dudanova et al., 2010), this growth factor likely activates local signaling that modulates the cytoskeleton in a manner similar to non-neuronal cells (Mulligan, 2018). Similar to other RTKs upon binding the GDNF-GFRα complex, Ret dimerizes and auto-phosphorylates multiple tyrosine residues, which recruit adaptor and signaling protein complexes (Mulligan, 2018). Ret receptors sustain local signaling by recruitment into lipid rafts containing caveolins, while non-compartmentalized Ret receptors are rapidly ubiquitinated by CBL family ligases and degraded (Pierchala et al., 2006). Adaptor proteins activate downstream signals involved in cytoskeletal dynamics, such as RAS-MAPK and PI3K-Akt signaling pathways. Coimmunoprecipitation experiments show that in response to GDNF treatment, Ret within lipid rafts interacts with actin filaments. Latrunculin B and jasplakinolide were used to disrupt or enhance actin polymerization, leading to impaired or enhanced translocation of Ret into lipid rafts, respectively, suggesting that F-actin is necessary for GDNF-induced cell signaling in mesencephalic dopaminergic cell lines (Li L. et al., 2017). Ret receptors within membrane microdomains also specifically interact with p60Src to promote neurite outgrowth and survival in cerebral granule cells. These effects depended on PI3K signaling, as treatment with LY294002, a PI3K inhibitor, prevented p60Src activation (Encinas et al., 2001). As discussed above, GDNF signals through NCAM/GFRα1 receptor complexes in CIs to modulate responses to Sema3B at the midline. Here GDNF treatment blocks Calpain-dependent cleavage of Plexin-A1 receptors, sensitizing post-crossing CIs to Sema3A (Charoy et al., 2012). However, much remains unknown about how GDNF induces rapid and local changes in growth cone motility and given the diverse population of neurons that express varied receptor complexes, focused research will be necessary to uncover how GDNF ligands precisely regulate axon guidance.

### Hepatocyte Growth Factor

Several studies have investigated the cooperative actions of HGF with other growth factors. While HGF has no outgrowth promoting activity on DRG neurons alone, it does enhance the neurotrophic effects of NGF in a dose-dependent manner on mouse DRG explant axon outgrowth (Maina et al., 1997). Similar cooperativity of HGF with NGF was also demonstrated with sympathetic neuron axon outgrowth and branching, where alone HGF has no effect, but when applied with NGF, it robustly enhances axon extension (Maina et al., 1998). However, in another study, it is curious to note that HGF applied as a gradient to DRG growth cones both stimulated the rate of axon extension and chemoattraction toward HGF (Sanford et al., 2008). In contrast to the cooperative effects described earlier, co-addition of NGF in the bath solution reversed the positive effects of HGF on DRG axon extension and blocked chemoattraction toward HGF (Sanford et al., 2008). The conditions used in these studies on DRG axon outgrowth, which may explain the observed discrepancies, are that the later study used acute and local stimulation with HGF, whereas other studies only tested the effects of chronic bath applied growth factors. In the future it will be important to test the signals activated by HGF in growth cones, as extensive evidence suggests that signals that regulate the cytoskeleton are activated downstream of c-MET receptors (Xiang et al., 2017). In any case, these studies effectively reveal the complexity of HGF driven axon guidance and the significant cross talk between RTK signaling mechanisms where tight regulation of cue exposure is likely required for proper neural connectivity.

### Insulin-Like Growth Factor

Neuronal growth cones express IGF RTKs on their surface (Quiroga et al., 1995; Ozdinler and Macklis, 2006), which are activated on axons of polarized hippocampal neurons in response to IGF-1 (Sosa et al., 2006). Further, IGF signaling is likely specifically enhanced in hippocampal axons by targeted insertion of new IGFRs and plasma membrane expansion through exocyst complex-mediated membrane fusion (Sosa et al., 2006). Delivery of non-synaptic vesicles containing IGFRs requires functional kinesin 2 and SNARE proteins (Morfini et al., 1997; Grassi et al., 2015). Similar to other GF receptors, PI3K/Akt and ERK/MAPK pathways function downstream of IGFR activation (Ozdinler and Macklis, 2006; Scolnick et al., 2008). In addition, IGF-1 treatment of human neuroblastoma cells results in rapid phosphorylation of IGF1R, followed by tyrosine phosphorylation of paxillin and focal adhesion kinase (FAK) coincident with lamellipodial advance (Leventhal and Feldman, 1996). Rapid phosphorylation of adhesion molecules downstream of growth factors and axon guidance cues have similarly been described in primary neurons (Robles and Gomez, 2006; Woo et al., 2009).

### Vascular Endothelial Growth Factor

Vascular endothelial growth factor activates several of the same signaling pathways as the growth factors discussed above that link to the cytoskeleton. For example, VEGF activates Src family kinases (SFKs) in CI growth cones as the Src inhibitor PP2 blocks VEGF-dependent chemoattraction (Ruiz de Almodovar et al., 2011). Similarly, VEGF activates SFKs in hippocampal axon growth cones and SFK activity is required downstream of VEGF for axon branch dynamics (Luck et al., 2019). In hippocampal dendrite branching, VEGFR2 endocytosis is necessary to activate both SFKs and Akt (Harde et al., 2019). It is interesting to note that VEGF-induced VEGFR2 internalization and spine maturation requires EphrinB2 receptors as VEGFR2/EphrinB2 compound heterozygous hippocampal neurons have reduced dendrite branching and spine size (Harde et al., 2019). VEGF treatment also triggers rapid redistribution and colocalization of cofilin and Arp2/3 complex to the actin cytoskeleton in chick DRG neuron growth cones. VEGF activation of cofilin and Arp2/3 promotes growth cone motility by these neurons (Schlau et al., 2018). VEGF-dependent Src activity not only appears to influence the cytoskeleton but regulates calcium influx through NMDA receptors. In cerebellar granule cells, VEGF promotes Flk1 clustering with NR2B subunits, Src-dependent tyrosine phosphorylation of NR2B and increased channel conductance (Meissirel et al., 2011). Together, extensive evidence demonstrates that VEGF acts on signaling pathways that modulate growth cone turning and neuronal morphogenesis, suggesting that VEGF functions as an essential axon guidance molecule for the developing nervous system.

## Conclusion

Extensive *in vivo* and *in vitro* evidence suggests that growth factors contribute to neural network assembly by regulating neuronal morphogenesis during development. First, growth factor receptors and ligands are properly distributed in embryonic neurons and target tissues across the CNS and PNS, supporting roles at intermediate choice points and final target destinations. Second, many knock-out studies performed *in vivo* strongly indicate roles for growth factors in network assembly (Kramer et al., 2006; Lopez-Bendito et al., 2006; Shirasaki et al., 2006; Chen et al., 2010; Erskine et al., 2011; Hancock et al., 2011; Wu et al., 2012). Finally, many studies performed *in vitro* show that growth factors can directly influence growth cone motility. *In vitro* studies have allowed further assessment of molecular mechanisms governing growth factor responses in simplified conditions. Defined conditions in culture will also enable us to better understand how classic axon guidance cues and common growth factors may interact with and modulate one another. This important problem is yet inaccessible *in vivo*, and we know that growth cones express multiple receptors and function as complex signal integrators (Dudanova and Klein, 2013). Crosstalk between growth factors and guidance cues likely occurs between most of the molecules discussed here, but further investigation is necessary to fully understand the modulatory roles of growth factors in axon guidance.

Mechanisms identified *in vitro* should ultimately be validated *in vivo*, as responses observed *in vitro* may not always match observations *in vivo.* Difference *in vitro* likely depends on how cues are presented to neurons in simplified culture conditions, compared to highly complex, multi-ligand, and mechanically variable conditions *in vivo*. However, these complexities *in vivo* should not discourage experiments designed to identify mechanisms in living organisms, as it is of paramount importance to understand how growth factors function *in vivo*. In addition, it is also important to examine sensitivities to growth factors and mechanisms of action in human neurons. Working with different classes of human neurons and non-neuronal cells has become possible with advances in stem cell differentiation techniques. One advantage here is that neurons carrying specific disease-causing mutations can be tested. Moreover, while working with human neurons *in vivo* is possible using xenografts (Linaro et al., 2019; Palma-Tortosa et al., 2020), recent advances in generating complex three-dimension human tissues and neural organoids from stem cells will make understanding mechanisms in more realistic *in vivo*-like conditions more feasible (Pasca, 2019). Using a combination of experimental model systems both *in vivo* and *in vitro* will allow us to one day clearly understand the detailed roles of each growth factor in neural network assembly.

## Author Contributions

TG provided the conceptual framework for this review. TG, MO, and CS wrote the manuscript. TC and SR provided editorial comments. All the authors contributed to the article and approved the submitted version.

## Conflict of Interest

The authors declare that the research was conducted in the absence of any commercial or financial relationships that could be construed as a potential conflict of interest.

## References

[B1] Abe Y. Namba H. Zheng Y. Nawa H. (2009). In situ hybridization reveals developmental regulation of ErbB1-4 mRNA expression in mouse midbrain: implication of ErbB receptors for dopaminergic neurons. *Neuroscience* 161 95–110. 10.1016/j.neuroscience.2009.03.022 19298847

[B2] Achim C. L. Katyal S. Wiley C. A. Shiratori M. Wang G. Oshika E. (1997). Expression of HGF and cMet in the developing and adult brain. *Brain Res. Dev. Brain Res.* 102 299–303. 10.1016/s0165-3806(97)00108-9 9352114

[B3] Adler R. Landa K. B. Manthorpe M. Varon S. (1979). Cholinergic neuronotrophic factors: intraocular distribution of trophic activity for ciliary neurons. *Science* 204 1434–1436. 10.1126/science.451576 451576

[B4] Aguirre A. Rubio M. E. Gallo V. (2010). Notch and EGFR pathway interaction regulates neural stem cell number and self-renewal. *Nature* 467 323–327. 10.1038/nature09347 20844536 PMC2941915

[B5] Airaksinen M. S. Holm L. Hatinen T. (2006). Evolution of the GDNF family ligands and receptors. *Brain Behav. Evol.* 68 181–190. 10.1159/000094087 16912471

[B6] Allard J. B. Duan C. (2018). IGF-binding proteins: why do they exist and why are there so many? *Front. Endocrinol.* 9:117. 10.3389/fendo.2018.00117 29686648 PMC5900387

[B7] Ando H. Luo Q. Koide N. Okada H. Urano A. (2006). Effects of insulin-like growth factor I on GnRH-induced gonadotropin subunit gene expressions in masu salmon pituitary cells at different stages of sexual maturation. *Gen. Comp. Endocrinol.* 149 21–29. 10.1016/j.ygcen.2006.04.013 16765954

[B8] Anton E. S. Ghashghaei H. T. Weber J. L. McCann C. Fischer T. M. Cheung I. D. (2004). Receptor tyrosine kinase ErbB4 modulates neuroblast migration and placement in the adult forebrain. *Nat. Neurosci.* 7 1319–1328. 10.1038/nn1345 15543145

[B9] Aoyagi A. Nishikawa K. Saito H. Abe K. (1994). Characterization of basic fibroblast growth factor-mediated acceleration of axonal branching in cultured rat hippocampal neurons. *Brain Res.* 661 117–126. 10.1016/0006-8993(94)91188-6 7834363

[B10] Apte R. S. Chen D. S. Ferrara N. (2019). VEGF in signaling and disease: beyond discovery and development. *Cell* 176 1248–1264. 10.1016/j.cell.2019.01.021 30849371 PMC6410740

[B11] Armelin H. A. (1973). Pituitary extracts and steroid hormones in the control of 3T3 cell growth. *Proc. Natl. Acad. Sci. U.S.A.* 70 2702–2706. 10.1073/pnas.70.9.2702 4354860 PMC427087

[B12] Askvig J. M. Watt J. A. (2015). The MAPK and PI3K pathways mediate CNTF-induced neuronal survival and process outgrowth in hypothalamic organotypic cultures. *J. Cell Commun. Signal.* 9 217–231. 10.1007/s12079-015-0268-8 25698661 PMC4580676

[B13] Balasubramanian R. Zhang X. (2016). Mechanisms of FGF gradient formation during embryogenesis. *Semin. Cell Dev. Biol.* 53 94–100. 10.1016/j.semcdb.2015.10.004 26454099 PMC4906438

[B14] Baldanzi G. Graziani A. (2014). Physiological signaling and structure of the HGF receptor MET. *Biomedicines* 3 1–31. 10.3390/biomedicines3010001 28536396 PMC5344233

[B15] Bao J. Lin H. Ouyang Y. Lei D. Osman A. Kim T. W. (2004). Activity-dependent transcription regulation of PSD-95 by neuregulin-1 and Eos. *Nat. Neurosci.* 7 1250–1258. 10.1038/nn1342 15494726

[B16] Beck K. D. Powell-Braxton L. Widmer H. R. Valverde J. Hefti F. (1995). Igf1 gene disruption results in reduced brain size, CNS hypomyelination, and loss of hippocampal granule and striatal parvalbumin-containing neurons. *Neuron* 14 717–730. 10.1016/0896-6273(95)90216-3 7718235

[B17] Bellon A. Luchino J. Haigh K. Rougon G. Haigh J. Chauvet S. (2010). VEGFR2 (KDR/Flk1) signaling mediates axon growth in response to semaphorin 3E in the developing brain. *Neuron* 66 205–219. 10.1016/j.neuron.2010.04.006 20434998

[B18] Beltran W. A. Zhang Q. Kijas J. W. Gu D. Rohrer H. Jordan J. A. (2003). Cloning, mapping, and retinal expression of the canine ciliary neurotrophic factor receptor alpha (CNTFRalpha). *Invest. Ophthalmol. Vis. Sci.* 44 3642–3649. 10.1167/iovs.02-0763 12882818

[B19] Bermingham-McDonogh O. McCabe K. L. Reh T. A. (1996). Effects of GGF/neuregulins on neuronal survival and neurite outgrowth correlate with erbB2/neu expression in developing rat retina. *Development* 122 1427–1438. 10.1242/dev.122.5.1427 8625831

[B20] Billings P. C. Pacifici M. (2015). Interactions of signaling proteins, growth factors and other proteins with heparan sulfate: mechanisms and mysteries. *Connect Tissue Res.* 56 272–280. 10.3109/03008207.2015.1045066 26076122 PMC4785798

[B21] Bonafina A. Fontanet P. A. Paratcha G. Ledda F. (2018). GDNF/GFRalpha1 complex abrogates self-renewing activity of cortical neural precursors inducing their differentiation. *Stem Cell Rep.* 10 1000–1015. 10.1016/j.stemcr.2018.01.019 29478900 PMC5918270

[B22] Bonafina A. Trinchero M. F. Rios A. S. Bekinschtein P. Schinder A. F. Paratcha G. (2019). GDNF and GFRalpha1 are required for proper integration of adult-born hippocampal neurons. *Cell Rep.* 29 4308.e4–4319.e4. 31875542 10.1016/j.celrep.2019.11.100

[B23] Bonanomi D. Chivatakarn O. Bai G. Abdesselem H. Lettieri K. Marquardt T. (2012). Ret is a multifunctional coreceptor that integrates diffusible- and contact-axon guidance signals. *Cell* 148 568–582. 10.1016/j.cell.2012.01.024 22304922 PMC3286831

[B24] Boscher C. Mege R. M. (2008). Cadherin-11 interacts with the FGF receptor and induces neurite outgrowth through associated downstream signalling. *Cell Signal* 20 1061–1072. 10.1016/j.cellsig.2008.01.008 18302981

[B25] Cahill M. E. Remmers C. Jones K. A. Xie Z. Sweet R. A. Penzes P. (2013). Neuregulin1 signaling promotes dendritic spine growth through kalirin. *J. Neurochem.* 126 625–635. 10.1111/jnc.12330 23742124 PMC3752328

[B26] Cariboni A. Davidson K. Dozio E. Memi F. Schwarz Q. Stossi F. (2011). VEGF signalling controls GnRH neuron survival via NRP1 independently of KDR and blood vessels. *Development* 138 3723–3733. 10.1242/dev.063362 21828096 PMC3152927

[B27] Caton A. Hacker A. Naeem A. Livet J. Maina F. Bladt F. (2000). The branchial arches and HGF are growth-promoting and chemoattractant for cranial motor axons. *Development* 127 1751–1766. 10.1242/dev.127.8.1751 10725250

[B28] Chang J. H. Gill S. Settleman J. Parsons S. J. (1995). c-Src regulates the simultaneous rearrangement of actin cytoskeleton, p190RhoGAP, and p120RasGAP following epidermal growth factor stimulation. *J. Cell Biol.* 130 355–368. 10.1083/jcb.130.2.355 7542246 PMC2199934

[B29] Charoy C. Nawabi H. Reynaud F. Derrington E. Bozon M. Wright K. (2012). gdnf activates midline repulsion by Semaphorin3B via NCAM during commissural axon guidance. *Neuron* 75 1051–1066. 10.1016/j.neuron.2012.08.021 22998873

[B30] Chen Y. Hancock M. L. Role L. W. Talmage D. A. (2010). Intramembranous valine linked to schizophrenia is required for neuregulin 1 regulation of the morphological development of cortical neurons. *J. Neurosci.* 30 9199–9208. 10.1523/jneurosci.0605-10.2010 20610754 PMC2919805

[B31] Chen Z. Donnelly C. R. Dominguez B. Harada Y. Lin W. Halim A. S. (2017). p75 is required for the establishment of postnatal sensory neuron diversity by potentiating ret signaling. *Cell Rep.* 21 707–720. 10.1016/j.celrep.2017.09.037 29045838 PMC5705950

[B32] Choi-Lundberg D. L. Bohn M. C. (1995). Ontogeny and distribution of glial cell line-derived neurotrophic factor (GDNF) mRNA in rat. *Brain Res. Dev. Brain Res.* 85 80–88. 10.1016/0165-3806(94)00197-8 7781171

[B33] Condron B. G. (1999). Serotonergic neurons transiently require a midline-derived FGF signal. *Neuron* 24 531–540. 10.1016/s0896-6273(00)81110-1 10595507

[B34] Cortes D. Carballo-Molina O. A. Castellanos-Montiel M. J. Velasco I. (2017). The non-survival effects of glial cell line-derived neurotrophic factor on neural cells. *Front. Mol. Neurosci.* 10:258. 10.3389/fnmol.2017.00258 28878618 PMC5572274

[B35] Csanaky K. Hess M. W. Klimaschewski L. (2019). Membrane-associated, not cytoplasmic or nuclear, FGFR1 induces neuronal differentiation. *Cells* 8:243. 10.3390/cells8030243 30875802 PMC6468866

[B36] Czarnek M. Bereta J. (2020). Proteolytic processing of neuregulin 2. *Mol. Neurobiol.* 57 1799–1813. 10.1007/s12035-019-01846-9 31838721 PMC7118043

[B37] Dang M. Dubbin K. D’Aiello A. Hartmann M. Lodish H. Herrlich A. (2011). Epidermal growth factor (EGF) ligand release by substrate-specific a disintegrin and metalloproteases (ADAMs) involves different protein kinase C (PKC) isoenzymes depending on the stimulus. *J. Biol. Chem*, 286 17704–17713. 10.1074/jbc.m110.187823 21454702 PMC3093846

[B38] Davis S. Aldrich T. H. Stahl N. Pan L. Taga T. Kishimoto T. (1993). LIFR beta and gp130 as heterodimerizing signal transducers of the tripartite CNTF receptor. *Science* 260 1805–1808. 10.1126/science.8390097 8390097

[B39] De Strooper B. Vassar R. Golde T. (2010). The secretases: enzymes with therapeutic potential in Alzheimer disease. *Nat. Rev. Neurol.* 6 99–107. 10.1038/nrneurol.2009.218 20139999 PMC2879045

[B40] DeChiara T. M. Efstratiadis A. Robertson E. J. (1990). A growth-deficiency phenotype in heterozygous mice carrying an insulin-like growth factor II gene disrupted by targeting. *Nature* 345 78–80. 10.1038/345078a0 2330056

[B41] DeChiara T. M. Vejsada R. Poueymirou W. T. Acheson A. Suri C. Conover J. C. (1995). Mice lacking the CNTF receptor, unlike mice lacking CNTF, exhibit profound motor neuron deficits at birth. *Cell* 83 313–322. 10.1016/0092-8674(95)90172-8 7585948

[B42] Decourtye L. Mire E. Clemessy M. Heurtier V. Ledent T. Robinson I. C. (2017). IGF-1 Induces GHRH neuronal axon elongation during early postnatal life in mice. *PLoS One* 12:e0170083. 10.1371/journal.pone.0170083 28076448 PMC5226784

[B43] Dolgin E. (2017). The most popular genes in the human genome. *Nature* 551 427–431. 10.1038/d41586-017-07291-9 29168817

[B44] Donnelly C. R. Shah A. A. Mistretta C. M. Bradley R. M. Pierchala B. A. (2018). Biphasic functions for the GDNF-Ret signaling pathway in chemosensory neuron development and diversification. *Proc. Natl. Acad. Sci. U.S.A.* 115 E516–E525. 29282324 10.1073/pnas.1708838115 PMC5776963

[B45] Donnelly C. R. Shah A. A. Suh E. B. Pierchala B. A. (2019). Ret signaling is required for tooth pulp innervation during organogenesis. *J. Dent. Res.* 98 705–712. 10.1177/0022034519837971 30958726 PMC6535917

[B46] Dudanova I. Gatto G. Klein R. (2010). GDNF acts as a chemoattractant to support ephrinA-induced repulsion of limb motor axons. *Curr. Biol.* 20 2150–2156. 10.1016/j.cub.2010.11.021 21109439

[B47] Dudanova I. Klein R. (2013). Integration of guidance cues: parallel signaling and crosstalk. *Trends Neurosci.* 36 295–304. 10.1016/j.tins.2013.01.007 23485451

[B48] Ebens A. Brose K. Leonardo E. D. Hanson M. G. Jr. Bladt F. Birchmeier C. (1996). Hepatocyte growth factor/scatter factor is an axonal chemoattractant and a neurotrophic factor for spinal motor neurons. *Neuron* 17 1157–1172. 10.1016/s0896-6273(00)80247-0 8982163

[B49] Eddy R. J. Weidmann M. D. Sharma V. P. Condeelis J. S. (2017). Tumor cell invadopodia: invasive protrusions that orchestrate metastasis. *Trends Cell Biol.* 27 595–607. 10.1016/j.tcb.2017.03.003 28412099 PMC5524604

[B50] Eichmann A. Thomas J. L. (2013). Molecular parallels between neural and vascular development. *Cold Spring Harb. Perspect. Med.* 3:a006551. 10.1101/cshperspect.a006551 23024177 PMC3530036

[B51] Encinas M. Tansey M. G. Tsui-Pierchala B. A. Comella J. X. Milbrandt J. Johnson E. M. Jr. (2001). c-Src is required for glial cell line-derived neurotrophic factor (GDNF) family ligand-mediated neuronal survival via a phosphatidylinositol-3 kinase (PI-3K)-dependent pathway. *J. Neurosci.* 21 1464–1472. 10.1523/jneurosci.21-05-01464.2001 11222636 PMC6762937

[B52] Erskine L. Reijntjes S. Pratt T. Denti L. Schwarz Q. Vieira J. M. (2011). VEGF signaling through neuropilin 1 guides commissural axon crossing at the optic chiasm. *Neuron* 70 951–965. 10.1016/j.neuron.2011.02.052 21658587 PMC3114076

[B53] Ferrara N. Carver-Moore K. Chen H. Dowd M. Lu L. O’Shea K. S. (1996). Heterozygous embryonic lethality induced by targeted inactivation of the VEGF gene. *Nature* 380 439–442. 10.1038/380439a0 8602242

[B54] Folkman J. Merler E. Abernathy C. Williams G. (1971). Isolation of a tumor factor responsible for angiogenesis. *J. Exp. Med.* 133 275–288. 10.1084/jem.133.2.275 4332371 PMC2138906

[B55] Gallagher D. Gutierrez H. Gavalda N. O’Keeffe G. Hay R. Davies A. M. (2007). Nuclear factor-kappaB activation via tyrosine phosphorylation of inhibitor kappaB-alpha is crucial for ciliary neurotrophic factor-promoted neurite growth from developing neurons. *J. Neurosci.* 27 9664–9669. 10.1523/jneurosci.0608-07.2007 17804627 PMC3512131

[B56] Garcia-Segura L. M. Perez J. Pons S. Rejas M. T. Torres-Aleman I. (1991). Localization of insulin-like growth factor I (IGF-I)-like immunoreactivity in the developing and adult rat brain. *Brain Res.* 560 167–174. 10.1016/0006-8993(91)91228-s 1722132

[B57] Gerecke K. M. Wyss J. M. Carroll S. L. (2004). Neuregulin-1beta induces neurite extension and arborization in cultured hippocampal neurons. *Mol. Cell Neurosci.* 27 379–393. 10.1016/j.mcn.2004.08.001 15555917

[B58] Giacobini P. Messina A. Wray S. Giampietro C. Crepaldi T. Carmeliet P. (2007). Hepatocyte growth factor acts as a motogen and guidance signal for gonadotropin hormone-releasing hormone-1 neuronal migration. *J. Neurosci.* 27 431–445. 10.1523/jneurosci.4979-06.2007 17215404 PMC6672060

[B59] Gomez T. M. Letourneau P. C. (2014). Actin dynamics in growth cone motility and navigation. *J. Neurochem.* 129 221–234. 10.1111/jnc.12506 24164353 PMC3980044

[B60] Grassi D. Plonka F. B. Oksdath M. Guil A. N. Sosa L. J. Quiroga S. (2015). Selected SNARE proteins are essential for the polarized membrane insertion of igf-1 receptor and the regulation of initial axonal outgrowth in neurons. *Cell Discov.* 1:15023. 27462422 10.1038/celldisc.2015.23 PMC4860833

[B61] Gregg C. Weiss S. (2005). CNTF/LIF/gp130 receptor complex signaling maintains a VZ precursor differentiation gradient in the developing ventral forebrain. *Development* 132 565–578. 10.1242/dev.01592 15634701

[B62] Guillemot F. Zimmer C. (2011). From cradle to grave: the multiple roles of fibroblast growth factors in neural development. *Neuron* 71 574–588. 10.1016/j.neuron.2011.08.002 21867876

[B63] Guthrie S. (2007). Neurotrophic factors: are they axon guidance molecules? *Adv. Exp. Med. Biol.* 621 81–94. 10.1007/978-0-387-76715-4_6 18269212

[B64] Gutierrez H. Dolcet X. Tolcos M. Davies A. (2004). HGF regulates the development of cortical pyramidal dendrites. *Development* 131 3717–3726. 10.1242/dev.01209 15229174

[B65] Hancock M. L. Nowakowski D. W. Role L. W. Talmage D. A. Flanagan J. G. (2011). Type III neuregulin 1 regulates pathfinding of sensory axons in the developing spinal cord and periphery. *Development* 138 4887–4898. 10.1242/dev.072306 22028026 PMC3201659

[B66] Harada T. Harada C. Kohsaka S. Wada E. Yoshida K. Ohno S. (2002). Microglia-Muller glia cell interactions control neurotrophic factor production during light-induced retinal degeneration. *J. Neurosci.* 22 9228–9236. 10.1523/jneurosci.22-21-09228.2002 12417648 PMC6758038

[B67] Harde E. Nicholson L. Furones Cuadrado B. Bissen D. Wigge S. Urban S. (2019). EphrinB2 regulates VEGFR2 during dendritogenesis and hippocampal circuitry development. *eLife* 8:e49819. 31868584 10.7554/eLife.49819 PMC6927743

[B68] Harris R. C. Chung E. Coffey R. J. (2003). EGF receptor ligands. *Exp. Cell Res.* 284 2–13. 10.1016/s0014-4827(02)00105-2 12648462

[B69] Hartnick C. J. Staecker H. Malgrange B. Lefebvre P. P. Liu W. Moonen G. (1996). Neurotrophic effects of BDNF and CNTF, alone and in combination, on postnatal day 5 rat acoustic ganglion neurons. *J. Neurobiol.* 30 246–254. 10.1002/(sici)1097-4695(199606)30:2<246::aid-neu6>3.0.co;2-5 8738753

[B70] Hazan R. B. Norton L. (1998). The epidermal growth factor receptor modulates the interaction of E-cadherin with the actin cytoskeleton. *J. Biol. Chem.* 273 9078–9084. 10.1074/jbc.273.15.9078 9535896

[B71] Hurtado-Chong A. Yusta-Boyo M. J. Vergano-Vera E. Bulfone A. de Pablo F. Vicario-Abejon C. (2009). IGF-I promotes neuronal migration and positioning in the olfactory bulb and the exit of neuroblasts from the subventricular zone. *Eur. J. Neurosci.* 30 742–755. 10.1111/j.1460-9568.2009.06870.x 19712103

[B72] Ip N. Y. McClain J. Barrezueta N. X. Aldrich T. H. Pan L. Li Y. (1993). The alpha component of the CNTF receptor is required for signaling and defines potential CNTF targets in the adult and during development. *Neuron* 10 89–102. 10.1016/0896-6273(93)90245-m 8381290

[B73] Ip N. Y. Nye S. H. Boulton T. G. Davis S. Taga T. Li Y. (1992). CNTF and LIF act on neuronal cells via shared signaling pathways that involve the IL-6 signal transducing receptor component gp130. *Cell* 69 1121–1132. 10.1016/0092-8674(92)90634-o 1617725

[B74] Irala D. Bonafina A. Fontanet P. A. Alsina F. C. Paratcha G. Ledda F. (2016). The GDNF-GFRalpha1 complex promotes the development of hippocampal dendritic arbors and spines via NCAM. *Development* 143 4224–4235. 10.1242/dev.140350 27707798

[B75] Isabella A. J. Barsh G. R. Stonick J. A. Dubrulle J. Moens C. B. (2020). Retinoic acid organizes the zebrafish vagus motor topographic map via spatiotemporal coordination of Hgf/Met signaling. *Dev. Cell* 53 344.e5–357.e5. 32302545 10.1016/j.devcel.2020.03.017 PMC7237105

[B76] Islam R. Kristiansen L. V. Romani S. Garcia-Alonso L. Hortsch M. (2004). Activation of EGF receptor kinase by L1-mediated homophilic cell interactions. *Mol. Biol. Cell* 15 2003–2012. 10.1091/mbc.e03-05-0333 14718570 PMC379294

[B77] Itoh N. Ornitz D. M. (2004). Evolution of the Fgf and Fgfr gene families. *Trends Genet.* 20 563–569. 10.1016/j.tig.2004.08.007 15475116

[B78] James J. M. Gewolb C. Bautch V. L. (2009). Neurovascular development uses VEGF-A signaling to regulate blood vessel ingression into the neural tube. *Development* 136 833–841. 10.1242/dev.028845 19176586 PMC2685948

[B79] Jones D. M. Tucker B. A. Rahimtula M. Mearow K. M. (2003). The synergistic effects of NGF and IGF-1 on neurite growth in adult sensory neurons: convergence on the PI 3-kinase signaling pathway. *J. Neurochem.* 86 1116–1128. 10.1046/j.1471-4159.2003.01925.x 12911620

[B80] Judson M. C. Bergman M. Y. Campbell D. B. Eagleson K. L. Levitt P. (2009). Dynamic gene and protein expression patterns of the autism-associated met receptor tyrosine kinase in the developing mouse forebrain. *J. Comp. Neurol.* 513 511–531. 10.1002/cne.21969 19226509 PMC2647986

[B81] Jung W. Castren E. Odenthal M. Vande Woude G. F. Ishii T. Dienes H. P. (1994). Expression and functional interaction of hepatocyte growth factor-scatter factor and its receptor c-met in mammalian brain. *J. Cell Biol.* 126 485–494. 10.1083/jcb.126.2.485 8034747 PMC2200035

[B82] Kato H. Wanaka A. Tohyama M. (1992). Co-localization of basic fibroblast growth factor-like immunoreactivity and its receptor mRNA in the rat spinal cord and the dorsal root ganglion. *Brain Res.* 576 351–354. 10.1016/0006-8993(92)90704-d 1325241

[B83] Kawamoto Y. Nakamura S. Matsuo A. Akiguchi I. Shibasaki H. (2000). Immunohistochemical localization of glial cell line-derived neurotrophic factor in the human central nervous system. *Neuroscience* 100 701–712. 10.1016/s0306-4522(00)00326-2 11036204

[B84] Keller S. Kneissl J. Grabher-Meier V. Heindl S. Hasenauer J. Maier D. (2017). Evaluation of epidermal growth factor receptor signaling effects in gastric cancer cell lines by detailed motility-focused phenotypic characterization linked with molecular analysis. *BMC Cancer* 17:845. 10.1186/s12885-017-3822-3 29237412 PMC5729506

[B85] Kenyon C. Chang J. Gensch E. Rudner A. Tabtiang R. (1993). A *C. elegans* mutant that lives twice as long as wild type. *Nature* 366 461–464. 10.1038/366461a0 8247153

[B86] Kipryushina Y. O. Yakovlev K. V. Odintsova N. A. (2015). Vascular endothelial growth factors: a comparison between invertebrates and vertebrates. *Cytokine Growth Factor Rev.* 26 687–695. 10.1016/j.cytogfr.2015.04.001 26066416

[B87] Kirsch M. Lee M. Y. Meyer V. Wiese A. Hofmann H. D. (1997). Evidence for multiple, local functions of ciliary neurotrophic factor (CNTF) in retinal development: expression of CNTF and its receptors and in vitro effects on target cells. *J. Neurochem.* 68 979–990. 10.1046/j.1471-4159.1997.68030979.x 9048743

[B88] Koprivica V. Cho K. S. Park J. B. Yiu G. Atwal J. Gore B. (2005). EGFR activation mediates inhibition of axon regeneration by myelin and chondroitin sulfate proteoglycans. *Science* 310 106–110. 10.1126/science.1115462 16210539

[B89] Kornblum H. I. Raymon H. K. Morrison R. S. Cavanaugh K. P. Bradshaw R. A. Leslie F. M. (1990). Epidermal growth factor and basic fibroblast growth factor: effects on an overlapping population of neocortical neurons in vitro. *Brain Res.* 535 255–263. 10.1016/0006-8993(90)91608-j 2073605

[B90] Kornblum H. I. Yanni D. S. Easterday M. C. Seroogy K. B. (2000). Expression of the EGF receptor family members ErbB2, ErbB3, and ErbB4 in germinal zones of the developing brain and in neurosphere cultures containing CNS stem cells. *Dev. Neurosci.* 22 16–24. 10.1159/000017423 10657694

[B91] Kramer E. R. Knott L. Su F. Dessaud E. Krull C. E. Helmbacher F. (2006). Cooperation between GDNF/Ret and ephrinA/EphA4 signals for motor-axon pathway selection in the limb. *Neuron* 50 35–47. 10.1016/j.neuron.2006.02.020 16600854

[B92] Leventhal P. S. Feldman E. L. (1996). Tyrosine phosphorylation and enhanced expression of paxillin during neuronal differentiation in vitro. *J. Biol. Chem.* 271 5957–5960. 10.1074/jbc.271.11.5957 8626373

[B93] Li C. Hisamoto N. Nix P. Kanao S. Mizuno T. Bastiani M. (2012). The growth factor SVH-1 regulates axon regeneration in *C. elegans* via the JNK MAPK cascade. *Nat. Neurosci.* 15 551–557. 10.1038/nn.3052 22388962

[B94] Li H. J. Sun Z. L. Yang X. T. Zhu L. Feng D. F. (2017). Exploring optic nerve axon regeneration. *Curr. Neuropharmacol.* 15 861–873. 28029073 10.2174/1570159X14666161227150250 PMC5652030

[B95] Li L. Song H. Mu P. Xu M. Liu C. Wang Y. (2017). The actin cytoskeleton is involved in glial cell line-derived neurotrophic factor (GDNF)-induced ret translocation into lipid rafts in dopaminergic neuronal cells. *Int. J. Mol. Sci.* 18:1922. 10.3390/ijms18091922 28880247 PMC5618571

[B96] Li S. Sato K. Gordon W. C. Sendtner M. Bazan N. G. Jin M. (2018). Ciliary neurotrophic factor (CNTF) protects retinal cone and rod photoreceptors by suppressing excessive formation of the visual pigments. *J. Biol. Chem.* 293 15256–15268. 10.1074/jbc.ra118.004008 30115683 PMC6166737

[B97] Li Y. Komuro Y. Fahrion J. K. Hu T. Ohno N. Fenner K. B. (2012). Light stimuli control neuronal migration by altering of insulin-like growth factor 1 (IGF-1) signaling. *Proc. Natl. Acad. Sci. U.S.A.* 109 2630–2635. 10.1073/pnas.1111326109 22308338 PMC3289354

[B98] Lin L. F. Doherty D. H. Lile J. D. Bektesh S. Collins F. (1993). GDNF: a glial cell line-derived neurotrophic factor for midbrain dopaminergic neurons. *Science* 260 1130–1132. 10.1126/science.8493557 8493557

[B99] Linaro D. Vermaercke B. Iwata R. Ramaswamy A. Libe-Philippot B. Boubakar L. (2019). Xenotransplanted human cortical neurons reveal species-specific development and functional integration into mouse visual circuits. *Neuron* 104 972.e6–986.e6. 31761708 10.1016/j.neuron.2019.10.002 PMC6899440

[B100] Lindsey S. Langhans S. A. (2015). Epidermal growth factor signaling in transformed cells. *Int. Rev. Cell Mol. Biol.* 314 1–41. 10.1016/bs.ircmb.2014.10.001 25619714 PMC4888053

[B101] Liu J. P. Baker J. Perkins A. S. Robertson E. J. Efstratiadis A. (1993). Mice carrying null mutations of the genes encoding insulin-like growth factor I (Igf-1) and type 1 IGF receptor (Igf1r). *Cell* 75 59–72. 10.1016/s0092-8674(05)80084-4 8402901

[B102] Lopez-Bendito G. Cautinat A. Sanchez J. A. Bielle F. Flames N. Garratt A. N. (2006). Tangential neuronal migration controls axon guidance: a role for neuregulin-1 in thalamocortical axon navigation. *Cell* 125 127–142. 10.1016/j.cell.2006.01.042 16615895 PMC2365888

[B103] Luck R. Urban S. Karakatsani A. Harde E. Sambandan S. Nicholson L. (2019). VEGF/VEGFR2 signaling regulates hippocampal axon branching during development. *eLife* 8:e49818. 31868583 10.7554/eLife.49818 PMC6927742

[B104] Lykissas M. G. Batistatou A. K. Charalabopoulos K. A. Beris A. E. (2007). The role of neurotrophins in axonal growth, guidance, and regeneration. *Curr. Neurovasc. Res.* 4 143–151. 10.2174/156720207780637216 17504212

[B105] MacInnis B. L. Campenot R. B. (2002). Retrograde support of neuronal survival without retrograde transport of nerve growth factor. *Science* 295 1536–1539. 10.1126/science.1064913 11799202

[B106] Mader C. C. Oser M. Magalhaes M. A. Bravo-Cordero J. J. Condeelis J. Koleske A. J. (2011). An EGFR-Src-Arg-cortactin pathway mediates functional maturation of invadopodia and breast cancer cell invasion. *Cancer Res.* 71 1730–1741. 10.1158/0008-5472.can-10-1432 21257711 PMC3057139

[B107] Maina F. Hilton M. C. Andres R. Wyatt S. Klein R. Davies A. M. (1998). Multiple roles for hepatocyte growth factor in sympathetic neuron development. *Neuron* 20 835–846. 10.1016/s0896-6273(00)80466-3 9620689

[B108] Maina F. Hilton M. C. Ponzetto C. Davies A. M. Klein R. (1997). Met receptor signaling is required for sensory nerve development and HGF promotes axonal growth and survival of sensory neurons. *Genes Dev.* 11 3341–3350. 10.1101/gad.11.24.3341 9407027 PMC316818

[B109] McFarlane S. Cornel E. Amaya E. Holt C. E. (1996). Inhibition of FGF receptor activity in retinal ganglion cell axons causes errors in target recognition. *Neuron* 17 245–254. 10.1016/s0896-6273(00)80156-7 8780648

[B110] McFarlane S. McNeill L. Holt C. E. (1995). FGF signaling and target recognition in the developing *Xenopus* visual system. *Neuron* 15 1017–1028. 10.1016/0896-6273(95)90091-8 7576646

[B111] Meissirel C. Ruiz de Almodovar C. Knevels E. Coulon C. Chounlamountri N. Segura I. (2011). VEGF modulates NMDA receptors activity in cerebellar granule cells through Src-family kinases before synapse formation. *Proc. Natl. Acad. Sci. U.S.A.* 108 13782–13787. 10.1073/pnas.1100341108 21804034 PMC3158143

[B112] Meyer D. Yamaai T. Garratt A. Riethmacher-Sonnenberg E. Kane D. Theill L. E. (1997). Isoform-specific expression and function of neuregulin. *Development* 124 3575–3586. 10.1242/dev.124.18.3575 9342050

[B113] Miller A. H. Howe H. B. Krause B. M. Friedle S. A. Banks M. I. Perkins B. D. (2018). Pregnancy-associated plasma protein-aa regulates photoreceptor synaptic development to mediate visually guided behavior. *J. Neurosci.* 38 5220–5236. 10.1523/jneurosci.0061-18.2018 29739870 PMC5977450

[B114] Miller R. G. Petajan J. H. Bryan W. W. Armon C. Barohn R. J. Goodpasture J. C. (1996). A placebo-controlled trial of recombinant human ciliary neurotrophic (rhCNTF) factor in amyotrophic lateral sclerosis. rhCNTF ALS Study Group. *Ann. Neurol.* 39 256–260. 10.1002/ana.410390215 8967757

[B115] Miwa K. Lee J. K. Takagishi Y. Opthof T. Fu X. Hirabayashi M. (2013). Axon guidance of sympathetic neurons to cardiomyocytes by glial cell line-derived neurotrophic factor (GDNF). *PLoS One* 8:e65202. 10.1371/journal.pone.0065202 23843937 PMC3701054

[B116] Modol-Caballero G. Santos D. Navarro X. Herrando-Grabulosa M. (2017). Neuregulin 1 reduces motoneuron cell death and promotes neurite growth in an in vitro model of motoneuron degeneration. *Front. Cell Neurosci.* 11:431. 10.3389/fncel.2017.00431 29375317 PMC5767462

[B117] Moore M. W. Klein R. D. Farinas I. Sauer H. Armanini M. Phillips H. (1996). Renal and neuronal abnormalities in mice lacking GDNF. *Nature* 382 76–79. 10.1038/382076a0 8657308

[B118] Morfini G. Quiroga S. Rosa A. Kosik K. Caceres A. (1997). Suppression of KIF2 in PC12 cells alters the distribution of a growth cone nonsynaptic membrane receptor and inhibits neurite extension. *J. Cell Biol.* 138 657–669. 10.1083/jcb.138.3.657 9245793 PMC2141628

[B119] Morrison R. S. Kornblum H. I. Leslie F. M. Bradshaw R. A. (1987). Trophic stimulation of cultured neurons from neonatal rat brain by epidermal growth factor. *Science* 238 72–75. 10.1126/science.3498986 3498986

[B120] Mouneimne G. DesMarais V. Sidani M. Scemes E. Wang W. Song X. (2006). Spatial and temporal control of cofilin activity is required for directional sensing during chemotaxis. *Curr. Biol.* 16 2193–2205. 10.1016/j.cub.2006.09.016 17113383

[B121] Mouneimne G. Soon L. DesMarais V. Sidani M. Song X. Yip S. C. (2004). Phospholipase C and cofilin are required for carcinoma cell directionality in response to EGF stimulation. *J. Cell Biol.* 166 697–708. 10.1083/jcb.200405156 15337778 PMC2172433

[B122] Moxham C. P. Duronio V. Jacobs S. (1989). Insulin-like growth factor I receptor beta-subunit heterogeneity. Evidence for hybrid tetramers composed of insulin-like growth factor I and insulin receptor heterodimers. *J. Biol. Chem.* 264 13238–13244. 2546949

[B123] Mulligan L. M. (2018). GDNF and the RET receptor in cancer: new insights and therapeutic potential. *Front. Physiol.* 9:1873. 10.3389/fphys.2018.01873 30666215 PMC6330338

[B124] Nakamura T. Teramoto H. Ichihara A. (1986). Purification and characterization of a growth factor from rat platelets for mature parenchymal hepatocytes in primary cultures. *Proc. Natl. Acad. Sci. U.S.A.* 83 6489–6493. 10.1073/pnas.83.17.6489 3529086 PMC386529

[B125] Nakano N. Kanekiyo K. Nakagawa T. Asahi M. Ide C. (2016). NTAK/neuregulin-2 secreted by astrocytes promotes survival and neurite outgrowth of neurons via ErbB3. *Neurosci. Lett.* 622 88–94. 10.1016/j.neulet.2016.04.050 27113200

[B126] Naldini L. Weidner K. M. Vigna E. Gaudino G. Bardelli A. Ponzetto C. (1991). Scatter factor and hepatocyte growth factor are indistinguishable ligands for the MET receptor. *EMBO J.* 10 2867–2878. 10.1002/j.1460-2075.1991.tb07836.x 1655405 PMC452997

[B127] Nassel D. R. Vanden Broeck J. (2016). Insulin/IGF signaling in *Drosophila* and other insects: factors that regulate production, release and post-release action of the insulin-like peptides. *Cell Mol. Life Sci.* 73 271–290. 10.1007/s00018-015-2063-3 26472340 PMC11108470

[B128] Nichol R. H. T. Catlett T. S. Onesto M. M. Hollender D. Gomez T. M. (2019). Environmental elasticity regulates cell-type specific RHOA signaling and neuritogenesis of human neurons. *Stem Cell Rep.* 13 1006–1021. 10.1016/j.stemcr.2019.10.008 31708476 PMC6915847

[B129] Nichols E. L. Smith C. J. (2019). Pioneer axons employ Cajal’s battering ram to enter the spinal cord. *Nat. Commun.* 10:562. 30718484 10.1038/s41467-019-08421-9 PMC6362287

[B130] Nieto-Estevez V. Defterali C. Vicario-Abejon C. (2016). IGF-I: a key growth factor that regulates neurogenesis and synaptogenesis from embryonic to adult stages of the brain. *Front. Neurosci.* 10:52. 10.3389/fnins.2016.00052 26941597 PMC4763060

[B131] O’Kusky J. Ye P. (2012). Neurodevelopmental effects of insulin-like growth factor signaling. *Front. Neuroendocrinol.* 33:230–251. 10.1016/j.yfrne.2012.06.002 22710100 PMC3677055

[B132] Olbrich L. Foehring D. Happel P. Brand-Saberi B. Theiss C. (2013). Fast rearrangement of the neuronal growth cone’s actin cytoskeleton following VEGF stimulation. *Histochem. Cell Biol.* 139 431–445. 10.1007/s00418-012-1036-y 23052841

[B133] Onuma T. A. Ding Y. Abraham E. Zohar Y. Ando H. Duan C. (2011). Regulation of temporal and spatial organization of newborn GnRH neurons by IGF signaling in zebrafish. *J. Neurosci.* 31 11814–11824. 10.1523/jneurosci.6804-10.2011 21849542 PMC6623180

[B134] Oo T. F. Ries V. Cho J. Kholodilov N. Burke R. E. (2005). Anatomical basis of glial cell line-derived neurotrophic factor expression in the striatum and related basal ganglia during postnatal development of the rat. *J. Comp. Neurol.* 484 57–67. 10.1002/cne.20463 15717300 PMC3092474

[B135] Oxvig C. (2015). The role of PAPP-A in the IGF system: location, location, location. *J. Cell Commun. Signal.* 9 177–187. 10.1007/s12079-015-0259-9 25617049 PMC4458251

[B136] Oyesiku N. M. Wigston D. J. (1996). Ciliary neurotrophic factor stimulates neurite outgrowth from spinal cord neurons. *J. Comp. Neurol.* 364 68–77. 10.1002/(sici)1096-9861(19960101)364:1<68::aid-cne6>3.0.co;2-q 8789276

[B137] Ozawa K. Uruno T. Miyakawa K. Seo M. Imamura T. (1996). Expression of the fibroblast growth factor family and their receptor family genes during mouse brain development. *Brain Res. Mol. Brain Res.* 41 279–288. 10.1016/0169-328x(96)00108-8 8883961

[B138] Ozdinler P. H. Macklis J. D. (2006). IGF-I specifically enhances axon outgrowth of corticospinal motor neurons. *Nat. Neurosci.* 9 1371–1381. 10.1038/nn1789 17057708

[B139] Pachnis V. Mankoo B. Costantini F. (1993). Expression of the c-ret proto-oncogene during mouse embryogenesis. *Development* 119 1005–1017. 10.1242/dev.119.4.1005 8306871

[B140] Page-McCaw A. Ewald A. J. Werb Z. (2007). Matrix metalloproteinases and the regulation of tissue remodelling. *Nat. Rev. Mol. Cell Biol.* 8 221–233. 17318226 10.1038/nrm2125 PMC2760082

[B141] Palma-Tortosa S. Tornero D. Gronning Hansen M. Monni E. Hajy M. Kartsivadze S. (2020). Activity in grafted human iPS cell-derived cortical neurons integrated in stroke-injured rat brain regulates motor behavior. *Proc. Natl. Acad. Sci. U.S.A.* 117 9094–9100. 10.1073/pnas.2000690117 32253308 PMC7183146

[B142] Paramo B. Wyatt S. Davies A. M. (2018). An essential role for neuregulin-4 in the growth and elaboration of developing neocortical pyramidal dendrites. *Exp. Neurol.* 302 85–92. 10.1016/j.expneurol.2018.01.002 29317193 PMC5866123

[B143] Paratcha G. Ibanez C. F. Ledda F. (2006). GDNF is a chemoattractant factor for neuronal precursor cells in the rostral migratory stream. *Mol. Cell Neurosci.* 31 505–514. 10.1016/j.mcn.2005.11.007 16380265

[B144] Paratcha G. Ledda F. (2008). GDNF and GFRalpha: a versatile molecular complex for developing neurons. *Trends Neurosci.* 31 384–391. 10.1016/j.tins.2008.05.003 18597864

[B145] Paratcha G. Ledda F. Ibanez C. F. (2003). The neural cell adhesion molecule NCAM is an alternative signaling receptor for GDNF family ligands. *Cell* 113 867–879. 10.1016/s0092-8674(03)00435-5 12837245

[B146] Pasca S. P. (2019). Assembling human brain organoids. *Science* 363 126–127. 10.1126/science.aau5729 30630918

[B147] Peterziel H. Unsicker K. Krieglstein K. (2002). TGFbeta induces GDNF responsiveness in neurons by recruitment of GFRalpha1 to the plasma membrane. *J. Cell Biol.* 159 157–167. 10.1083/jcb.200203115 12370242 PMC2173495

[B148] Pierchala B. A. Milbrandt J. Johnson E. M. Jr. (2006). Glial cell line-derived neurotrophic factor-dependent recruitment of Ret into lipid rafts enhances signaling by partitioning Ret from proteasome-dependent degradation. *J. Neurosci.* 26 2777–2787. 10.1523/jneurosci.3420-05.2006 16525057 PMC6675173

[B149] Platta H. W. Stenmark H. (2011). Endocytosis and signaling. *Curr. Opin. Cell Biol.* 23 393–403. 21474295 10.1016/j.ceb.2011.03.008

[B150] Povlsen G. K. Berezin V. Bock E. (2008). Neural cell adhesion molecule-180-mediated homophilic binding induces epidermal growth factor receptor (EGFR) down-regulation and uncouples the inhibitory function of EGFR in neurite outgrowth. *J. Neurochem.* 104 624–639. 17995934 10.1111/j.1471-4159.2007.05033.x

[B151] Pujic Z. Giacomantonio C. E. Unni D. Rosoff W. J. Goodhill G. J. (2008). Analysis of the growth cone turning assay for studying axon guidance. *J. Neurosci. Methods* 170 220–228. 10.1016/j.jneumeth.2008.01.014 18313760

[B152] Qiu S. Lu Z. Levitt P. (2014). MET receptor tyrosine kinase controls dendritic complexity, spine morphogenesis, and glutamatergic synapse maturation in the hippocampus. *J. Neurosci.* 34 16166–16179. 10.1523/jneurosci.2580-14.2014 25471559 PMC4252539

[B153] Quiroga S. Garofalo R. S. Pfenninger K. H. (1995). Insulin-like growth factor I receptors of fetal brain are enriched in nerve growth cones and contain a beta-subunit variant. *Proc. Natl. Acad. Sci. U.S.A.* 92 4309–4312. 10.1073/pnas.92.10.4309 7753803 PMC41933

[B154] Rahman-Enyart A. Lai C. Prieto A. L. (2020). Neuregulins 1, 2, and 3 promote early neurite outgrowth in ErbB4-Expressing cortical GABAergic interneurons. *Mol. Neurobiol.* 57 3568–3588. 10.1007/s12035-020-01966-7 32542595

[B155] Recio-Pinto E. Rechler M. M. Ishii D. N. (1986). Effects of insulin, insulin-like growth factor-II, and nerve growth factor on neurite formation and survival in cultured sympathetic and sensory neurons. *J. Neurosci.* 6 1211–1219. 10.1523/jneurosci.06-05-01211.1986 3519887 PMC6568556

[B156] Robles E. Gomez T. M. (2006). Focal adhesion kinase signaling at sites of integrin-mediated adhesion controls axon pathfinding. *Nat. Neurosci.* 9 1274–1283. 10.1038/nn1762 16964253

[B157] Rosenberg A. Noble E. P. (1989). EGF-induced neuritogenesis and correlated synthesis of plasma membrane gangliosides in cultured embryonic chick CNS neurons. *J. Neurosci. Res.* 24 531–536. 10.1002/jnr.490240411 2600976

[B158] Ruiz de Almodovar C. Coulon C. Salin P. A. Knevels E. Chounlamountri N. Poesen K. (2010). Matrix-binding vascular endothelial growth factor (VEGF) isoforms guide granule cell migration in the cerebellum via VEGF receptor Flk1. *J. Neurosci.* 30 15052–15066. 10.1523/jneurosci.0477-10.2010 21068311 PMC6633861

[B159] Ruiz de Almodovar C. Fabre P. J. Knevels E. Coulon C. Segura I. Haddick P. C. (2011). VEGF mediates commissural axon chemoattraction through its receptor Flk1. *Neuron* 70 966–978. 10.1016/j.neuron.2011.04.014 21658588 PMC3638787

[B160] Sahin U. Weskamp G. Kelly K. Zhou H. M. Higashiyama S. Peschon J. (2004). Distinct roles for ADAM10 and ADAM17 in ectodomain shedding of six EGFR ligands. *J. Cell Biol.* 164 769–779. 10.1083/jcb.200307137 14993236 PMC2172154

[B161] Salie R. Steeves J. D. (2005). IGF-1 and BDNF promote chick bulbospinal neurite outgrowth in vitro. *Int. J. Dev. Neurosci.* 23 587–598. 10.1016/j.ijdevneu.2005.07.003 16143487

[B162] Sanford S. D. Gatlin J. C. Hokfelt T. Pfenninger K. H. (2008). Growth cone responses to growth and chemotropic factors. *Eur. J. Neurosci.* 28 268–278. 10.1111/j.1460-9568.2008.06327.x 18702698

[B163] Santiago-Medina M. Gregus K. A. Nichol R. H. O’Toole S. M. Gomez T. M. (2015). Regulation of ECM degradation and axon guidance by growth cone invadosomes. *Development* 142 486–496. 10.1242/dev.108266 25564649 PMC4302990

[B164] Schafer K. H. Mestres P. (1999). The GDNF-induced neurite outgrowth and neuronal survival in dissociated myenteric plexus cultures of the rat small intestine decreases postnatally. *Exp. Brain Res.* 125 447–452. 10.1007/s002210050702 10323291

[B165] Schlau M. Terheyden-Keighley D. Theis V. Mannherz H. G. Theiss C. (2018). VEGF Triggers the activation of cofilin and the Arp2/3 complex within the growth cone. *Int. J. Mol. Sci.* 19:384. 10.3390/ijms19020384 29382077 PMC5855606

[B166] Schofer C. Frei K. Weipoltshammer K. Wachtler F. (2001). The apical ectodermal ridge, fibroblast growth factors (FGF-2 and FGF-4) and insulin-like growth factor I (IGF-I) control the migration of epidermal melanoblasts in chicken wing buds. *Anat. Embryol.* 203 137–146. 10.1007/s004290000148 11218060

[B167] Schuster K. Dambly-Chaudiere C. Ghysen A. (2010). Glial cell line-derived neurotrophic factor defines the path of developing and regenerating axons in the lateral line system of zebrafish. *Proc. Natl. Acad. Sci. U.S.A.* 107 19531–19536. 10.1073/pnas.1002171107 20974953 PMC2984216

[B168] Schwieger J. Warnecke A. Lenarz T. Esser K. H. Scheper V. (2015). Neuronal survival, morphology and outgrowth of spiral ganglion neurons using a defined growth factor combination. *PLoS One* 10:e0133680. 10.1371/journal.pone.0133680 26263175 PMC4532470

[B169] Scolnick J. A. Cui K. Duggan C. D. Xuan S. Yuan X. B. Efstratiadis A. (2008). Role of IGF signaling in olfactory sensory map formation and axon guidance. *Neuron* 57 847–857. 10.1016/j.neuron.2008.01.027 18367086 PMC2364597

[B170] Selvaraj B. T. Frank N. Bender F. L. Asan E. Sendtner M. (2012). Local axonal function of STAT3 rescues axon degeneration in the pmn model of motoneuron disease. *J. Cell Biol.* 199 437–451. 10.1083/jcb.201203109 23109669 PMC3483126

[B171] Sendtner M. Carroll P. Holtmann B. Hughes R. A. Thoenen H. (1994). Ciliary neurotrophic factor. *J. Neurobiol.* 25 1436–1453. 7852996 10.1002/neu.480251110

[B172] Shanmugalingam S. Houart C. Picker A. Reifers F. Macdonald R. Barth A. (2000). Ace/Fgf8 is required for forebrain commissure formation and patterning of the telencephalon. *Development* 127 2549–2561. 10.1242/dev.127.12.2549 10821754

[B173] Shirasaki R. Lewcock J. W. Lettieri K. Pfaff S. L. (2006). FGF as a target-derived chemoattractant for developing motor axons genetically programmed by the LIM code. *Neuron* 50 841–853. 10.1016/j.neuron.2006.04.030 16772167

[B174] Short C. A. Onesto M. M. Rempel S. K. Catlett T. S. Gomez T. M. (2021). Familiar growth factors have diverse roles in neural network assembly. *Curr. Opin. Neurobiol.* 66 233–239. 10.1016/j.conb.2020.12.016 33477094 PMC8058242

[B175] Sleeman M. W. Anderson K. D. Lambert P. D. Yancopoulos G. D. Wiegand S. J. (2000). The ciliary neurotrophic factor and its receptor, CNTFR alpha. *Pharm. Acta Helv.* 74 265–272. 10812968 10.1016/s0031-6865(99)00050-3

[B176] Smith T. G. Karlsson M. Lunn J. S. Eblaghie M. C. Keenan I. D. Farrell E. R. (2006). Negative feedback predominates over cross-regulation to control ERK MAPK activity in response to FGF signalling in embryos. *FEBS Lett.* 580 4242–4245. 10.1016/j.febslet.2006.06.081 16831426

[B177] Sondell M. Lundborg G. Kanje M. (1999). Vascular endothelial growth factor has neurotrophic activity and stimulates axonal outgrowth, enhancing cell survival and Schwann cell proliferation in the peripheral nervous system. *J. Neurosci.* 19 5731–5740. 10.1523/jneurosci.19-14-05731.1999 10407014 PMC6783109

[B178] Sosa L. Dupraz S. Laurino L. Bollati F. Bisbal M. Caceres A. (2006). IGF-1 receptor is essential for the establishment of hippocampal neuronal polarity. *Nat. Neurosci.* 9 993–995. 10.1038/nn1742 16845384

[B179] Srinivasan P. Zervantonakis I. K. Kothapalli C. R. (2014). Synergistic effects of 3D ECM and chemogradients on neurite outgrowth and guidance: a simple modeling and microfluidic framework. *PLoS One* 9:e99640. 10.1371/journal.pone.0099640 24914812 PMC4051856

[B180] Stahl N. Yancopoulos G. D. (1994). The tripartite CNTF receptor complex: activation and signaling involves components shared with other cytokines. *J. Neurobiol.* 25 1454–1466. 10.1002/neu.480251111 7852997

[B181] Stockli K. A. Lillien L. E. Naher-Noe M. Breitfeld G. Hughes R. A. Raff M. C. (1991). Regional distribution, developmental changes, and cellular localization of CNTF-mRNA and protein in the rat brain. *J. Cell Biol.* 115 447–459. 10.1083/jcb.115.2.447 1918150 PMC2289163

[B182] Stoker M. Perryman M. (1985). An epithelial scatter factor released by embryo fibroblasts. *J. Cell Sci.* 77 209–223. 10.1242/jcs.77.1.209 3841349

[B183] Stromberg I. Bjorklund L. Johansson M. Tomac A. Collins F. Olson L. (1993). Glial cell line-derived neurotrophic factor is expressed in the developing but not adult striatum and stimulates developing dopamine neurons in vivo. *Exp. Neurol.* 124 401–412. 10.1006/exnr.1993.1214 7904571

[B184] Syed N. Richardson P. Bulloch A. (1996). Ciliary neurotrophic factor, unlike nerve growth factor, supports neurite outgrowth but not synapse formation by adult *Lymnaea* neurons. *J. Neurobiol.* 29 293–303. 10.1002/(sici)1097-4695(199603)29:3<293::aid-neu2>3.0.co;2-4 8907159

[B185] Szebenyi G. Dent E. W. Callaway J. L. Seys C. Lueth H. Kalil K. (2001). Fibroblast growth factor-2 promotes axon branching of cortical neurons by influencing morphology and behavior of the primary growth cone. *J. Neurosci.* 21 3932–3941. 10.1523/jneurosci.21-11-03932.2001 11356881 PMC6762708

[B186] Talbott J. F. Cao Q. Bertram J. Nkansah M. Benton R. L. Lavik E. (2007). CNTF promotes the survival and differentiation of adult spinal cord-derived oligodendrocyte precursor cells in vitro but fails to promote remyelination in vivo. *Exp. Neurol.* 204 485–489. 10.1016/j.expneurol.2006.12.013 17274982 PMC2430994

[B187] Thewke D. P. Seeds N. W. (1996). Expression of hepatocyte growth factor/scatter factor, its receptor, c-met, and tissue-type plasminogen activator during development of the murine olfactory system. *J. Neurosci.* 16 6933–6944. 10.1523/jneurosci.16-21-06933.1996 8824331 PMC6579244

[B188] Tillo M. Erskine L. Cariboni A. Fantin A. Joyce A. Denti L. (2015). VEGF189 binds NRP1 and is sufficient for VEGF/NRP1-dependent neuronal patterning in the developing brain. *Development* 142 314–319. 10.1242/dev.115998 25519242 PMC4302834

[B189] Trejo J. L. Piriz J. Llorens-Martin M. V. Fernandez A. M. Bolos M. LeRoith D. (2007). Central actions of liver-derived insulin-like growth factor I underlying its pro-cognitive effects. *Mol. Psychiatry* 12 1118–1128. 10.1038/sj.mp.4002076 17848918

[B190] Trupp M. Belluardo N. Funakoshi H. Ibanez C. F. (1997). Complementary and overlapping expression of glial cell line-derived neurotrophic factor (GDNF), c-ret proto-oncogene, and GDNF receptor-alpha indicates multiple mechanisms of trophic actions in the adult rat CNS. *J. Neurosci.* 17 3554–3567. 10.1523/jneurosci.17-10-03554.1997 9133379 PMC6573713

[B191] Tsai N. P. Tsui Y. C. Pintar J. E. Loh H. H. Wei L. N. (2010). Kappa opioid receptor contributes to EGF-stimulated neurite extension in development. *Proc. Natl. Acad. Sci. U.S.A.* 107 3216–3221. 10.1073/pnas.0912367107 20133770 PMC2840278

[B192] Vaccarino F. M. Schwartz M. L. Raballo R. Nilsen J. Rhee J. Zhou M. (1999). Changes in cerebral cortex size are governed by fibroblast growth factor during embryogenesis. *Nat. Neurosci.* 2:848. 10.1038/12226 10461229

[B193] Vullhorst D. Ahmad T. Karavanova I. Keating C. Buonanno A. (2017). Structural similarities between neuregulin 1-3 isoforms determine their subcellular distribution and signaling mode in central neurons. *J. Neurosci.* 37 5232–5249. 10.1523/jneurosci.2630-16.2017 28432142 PMC5456106

[B194] Wahlin K. J. Lim L. Grice E. A. Campochiaro P. A. Zack D. J. Adler R. (2004). A method for analysis of gene expression in isolated mouse photoreceptor and Muller cells. *Mol. Vis.* 10 366–375. 15205663

[B195] Walicke P. Cowan W. M. Ueno N. Baird A. Guillemin R. (1986). Fibroblast growth factor promotes survival of dissociated hippocampal neurons and enhances neurite extension. *Proc. Natl. Acad. Sci. U.S.A.* 83 3012–3016. 10.1073/pnas.83.9.3012 3458259 PMC323437

[B196] Wang Q. Marcucci F. Cerullo I. Mason C. (2016). Ipsilateral and contralateral retinal ganglion cells express distinct genes during decussation at the optic chiasm. *eNeuro* 3:ENEURO.0169–16.2016. 10.1523/ENEURO.0169-16.2016 27957530 PMC5136615

[B197] Webber C. A. Hyakutake M. T. McFarlane S. (2003). Fibroblast growth factors redirect retinal axons in vitro and in vivo. *Dev. Biol.* 263 24–34. 10.1016/s0012-1606(03)00435-4 14568544

[B198] Williams E. J. Furness J. Walsh F. S. Doherty P. (1994). Activation of the FGF receptor underlies neurite outgrowth stimulated by L1, N-CAM, and N-cadherin. *Neuron* 13 583–594. 10.1016/0896-6273(94)90027-2 7917292

[B199] Wolman M. A. Jain R. A. Marsden K. C. Bell H. Skinner J. Hayer K. E. (2015). A genome-wide screen identifies PAPP-AA-mediated IGFR signaling as a novel regulator of habituation learning. *Neuron* 85 1200–1211. 10.1016/j.neuron.2015.02.025 25754827 PMC4368495

[B200] Woo S. Rowan D. J. Gomez T. M. (2009). Retinotopic mapping requires focal adhesion kinase-mediated regulation of growth cone adhesion. *J. Neurosci.* 29 13981–13991. 10.1523/jneurosci.4028-09.2009 19890008 PMC2796108

[B201] Wrighton K. H. (2019). Rules of invasion. *Nat. Rev. Neurosci.* 20 574–575. 10.1038/s41583-019-0215-4 31413334

[B202] Wrigley S. Arafa D. Tropea D. (2017). Insulin-like growth factor 1: at the crossroads of brain development and aging. *Front. Cell Neurosci.* 11:14. 10.3389/fncel.2017.00014 28203146 PMC5285390

[B203] Wu Q. F. Yang L. Li S. Wang Q. Yuan X. B. Gao X. (2012). Fibroblast growth factor 13 is a microtubule-stabilizing protein regulating neuronal polarization and migration. *Cell* 149 1549–1564. 10.1016/j.cell.2012.04.046 22726441

[B204] Xiang C. Chen J. Fu P. (2017). HGF/Met signaling in cancer invasion: the impact on cytoskeleton remodeling. *Cancers* 9:44. 10.3390/cancers9050044 28475121 PMC5447954

[B205] Xiang Y. Ding N. Xing Z. Zhang W. Liu H. Li Z. (2011). Insulin-like growth factor-1 regulates neurite outgrowth and neuronal migration from organotypic cultured dorsal root ganglion. *Int. J. Neurosci.* 121 101–106. 10.3109/00207454.2010.535935 21110707

[B206] Xiang Y. Y. Dong H. Wan Y. Li J. Yee A. Yang B. B. (2006). Versican G3 domain regulates neurite growth and synaptic transmission of hippocampal neurons by activation of epidermal growth factor receptor. *J. Biol. Chem.* 281 19358–19368. 10.1074/jbc.m512980200 16648628

[B207] Yang J. F. Zhou H. Choi R. C. Ip N. Y. Peng H. B. Tsim K. W. (1999). A cysteine-rich form of *Xenopus* neuregulin induces the expression of acetylcholine receptors in cultured myotubes. *Mol. Cell Neurosci.* 13 415–429. 10.1006/mcne.1999.0759 10383827

[B208] Zechel S. Fernandez-Suarez D. Ibanez C. F. (2018). Cell-autonomous role of GFRalpha1 in the development of olfactory bulb GABAergic interneurons. *Biol. Open* 7:bio033753. 29716946 10.1242/bio.033753 PMC5992528

[B209] Zhang F. Zheng L. Cheng S. Peng Y. Fu L. Zhang X. (2019). Comparison of the interactions of different growth factors and glycosaminoglycans. *Molecules* 24:3360. 10.3390/molecules24183360 31527407 PMC6767211

[B210] Zhou F. Q. Snider W. D. (2006). Intracellular control of developmental and regenerative axon growth. *Philos. Trans. R. Soc. Lond. B Biol. Sci.* 361 1575–1592. 10.1098/rstb.2006.1882 16939976 PMC1664665

